# Comparative analysis of 163 ant genomes reveals recurrent horizontal gene transfer from bacteria to ants

**DOI:** 10.1093/gigascience/giag043

**Published:** 2026-04-06

**Authors:** Janina L Rinke, Lukas Franke, Ding He, Maike L Fischer, Joel Vizueta, Lars A Eicholt, Rasmus S Larsen, Zijun Xiong, Phoebe Cunningham, Lee M Henry, Martin Kaltenpoth, Jürgen Gadau, Guojie Zhang, Jacobus J Boomsma, Lukas Schrader

**Affiliations:** Institute for Evolution and Biodiversity, University of Münster, DE-48149 Münster, Germany; Institute for Evolution and Biodiversity, University of Münster, DE-48149 Münster, Germany; Section for Ecology and Evolution, Department of Biology, University of Copenhagen, DK-2100 Copenhagen, Denmark; Department of Insect Symbiosis, Max Planck Institute for Chemical Ecology, DE-07745 Jena, Germany; Institute for Insect Biotechnology, Justus Liebig University of Gießen, DE-35392 Gießen, Germany; Section for Ecology and Evolution, Department of Biology, University of Copenhagen, DK-2100 Copenhagen, Denmark; Institute for Evolution and Biodiversity, University of Münster, DE-48149 Münster, Germany; Section for Ecology and Evolution, Department of Biology, University of Copenhagen, DK-2100 Copenhagen, Denmark; School of Basic Medical Sciences, Jiangxi Medical College, Nanchang University, China; School of Biological and Behavioural Sciences, Queen Mary University London, London E1 4NS, UK; School of Biological and Behavioural Sciences, Queen Mary University London, London E1 4NS, UK; Department of Insect Symbiosis, Max Planck Institute for Chemical Ecology, DE-07745 Jena, Germany; Institute for Evolution and Biodiversity, University of Münster, DE-48149 Münster, Germany; Evolutionary & Organismal Biology Research Center, Zhejiang University School of Medicine, Hangzhou 310058, China; Villum Centre for Biodiversity Genomics, Section for Ecology and Evolution, Department of Biology, University of Copenhagen, DK-2100 Copenhagen, Denmark; Women’s Hospital, School of Medicine, Zhejiang University, Shangcheng District, Hangzhou 310006, China; Section for Ecology and Evolution, Department of Biology, University of Copenhagen, DK-2100 Copenhagen, Denmark; Institute for Evolution and Biodiversity, University of Münster, DE-48149 Münster, Germany

**Keywords:** horizontal gene transfer, comparative genomics, Formicidae, social insects, bacteria, endosymbionts, lysozyme, peptidoglycan degradation

## Abstract

**Background:**

Horizontal gene transfer (HGT) from bacteria can drive phenotypic innovation and adaptation in eukaryotes. Ants are likely carriers of HGT-derived genes, as they have repeatedly established mutualistic associations with vertically transmitted bacterial symbionts with direct access to the germline. However, the prevalence of HGT across ants and most other insects remains virtually unexplored.

**Results:**

Here, we systematically investigated the genomes of over 160 species of ants and uncovered 497 protein-coding HGT events in 85 species, predominantly derived from intracellular symbionts. Among these, we identified several HGTs likely underpinning functional innovations, primarily by mediating immune-system adaptations or facilitating nutritional niche expansions. Several of these HGTs were conserved in sequence and synteny across multiple species, consistent with strong signatures of purifying selection over up to 40 million years. Functional and structural analysis of a horizontally acquired Xanthine-guanine phosphoribosyltransferase gene of *Cardiocondyla* ants reveals deep entrenchment of this protein in basic energy metabolism of the host, facilitated by the enzyme’s substrate promiscuity.

**Conclusions:**

This study provides insights into the abundance and diversity of HGT from bacteria in the evolutionary history of ants. Furthermore, our comparative and functional analyses suggest that many of the horizontally acquired genes serve adaptive functions in ants, most prominently by expanding metabolic pathways or modulating immune responses.

## Introduction

Horizontal gene transfer (HGT) between unrelated genomes is a key driver of evolutionary change, provided such transfers result in gene acquisition that natural selection can act on [1–3]. HGT between prokaryotic species has been extensively studied for the adaptive innovations it enabled, such as the spread of antibiotic resistance across species boundaries [4]. While HGT among prokaryotes is often reciprocal and well documented, growing evidence also highlights transfers from bacteria, fungi, or viruses toward multicellular eukaryotes, a process that may be promoted by the intimate associations between endosymbionts and their hosts [5–9]. These transfers appear asymmetrical because very few eukaryote genes are known to have become established in prokaryotes [5]. Owing to recent advances in genomics and molecular biology, systematic comparative analyses of HGT in multicellular eukaryotes have now become feasible [10–12]. Recent bioinformatics frameworks have further expanded the ability to systematically assess gene spread and horizontal gene transfer across microbial communities by integrating comparative genomics, functional annotation, and transfer detection [13]. However, in such studies it is crucial to realize that HGT events are functionally comparable to random macromutations and can only result in lasting phenotypic effects once they become subject to natural selection. Genome-wide screens of HGT in multicellular organisms should therefore focus on identifying which transferred elements have been maintained by natural selection, rather than simply persisting in the genome as neutral or slightly deleterious insertions that survived by genetic drift.

Until recently, prokaryote-to-eukaryote HGT has been controversial [14], as bacterial contaminations, ancestral genes lost from related extant lineages, or incorrect phylogenetic inferences have posed challenges for the correct identification of such putative HGT events [15]. Nonetheless, an expanding body of evidence indicates the widespread occurrence of HGTs across eukaryotic lineages, with a small subset shown to possess adaptive potential, substantiated by functional validation in specific taxa [16–18]. For example, at least 1,400 genetic elements from non-metazoan donors have recently been identified as part of insect genomes, of which one HGT has been further characterized and highlighted to play a role in male courtship behavior in lepidopterans [11]. Apart from that, functional HGTs in eukaryotes have been implied to mediate nutritional and metabolic diversification [8, 19, 20], as well as adaptive immune-system responses or previously undescribed antibacterial capacity [16, 17, 21–23], and to promote parasitism ability [24, 25].

Successful HGT requires integration of bacterial genetic material into the metazoan germline, which implies that vertically transmitted endosymbionts were most likely to act as donors. Such endosymbionts occur more commonly in some animal groups than in others, likely explaining why HGTs occurred more often in insects [26] than in vertebrates [27, 28]. Vertically transmitted endosymbionts such as *Wolbachia* or *Candidatus Blochmanniella* (formerly *Blochmannia*) are widespread within the ants [29, 30], an ecologically highly diverse and exclusively social insect family with over 15,000 described species. Some of these obligate endosymbionts have been vertically co-transmitted with their ant hosts for millions of years [31], making these intricate relationships likely sources for HGT events. However, in-depth comparative studies to elucidate the prevalence of HGTs in ants are still lacking.

In this study, we comprehensively searched for HGTs across 163 ant genomes that were recently subjected to general analyses [32]. This large-scale approach extends previous coverage by at least an order of magnitude, because HGTs have so far only been described in detail for two ant species, the wood ant *Formica exsecta* [33] and the heart-node ant *Cardiocondyla obscurior* [34]. In *F. exsecta*, multiple putative genes encoding ankyrin repeat domain (ANK) proteins, DNA repair proteins, and transposases have been identified as HGTs deriving from *Wolbachia* [33], while an HGT from *Candidatus Blochmanniella*-like enterobacteria has been described for *C. obscurior* [34]. Another recent study across 218 insect genomes identified putative HGTs in 20 ant species, but no effort was made to describe these in any detail [11]. Here, we identify and characterize 497 HGTs across 85 ant species, covering eight of the 17 extant ant subfamilies. Focusing on the most striking cases, we further provide in-depth analyses of the potential impact of HGTs on adaptive evolutionary processes in the ants.

## Results

### Ants acquired genes from bacteria on a large scale via horizontal gene transfer

**Figure 1:**
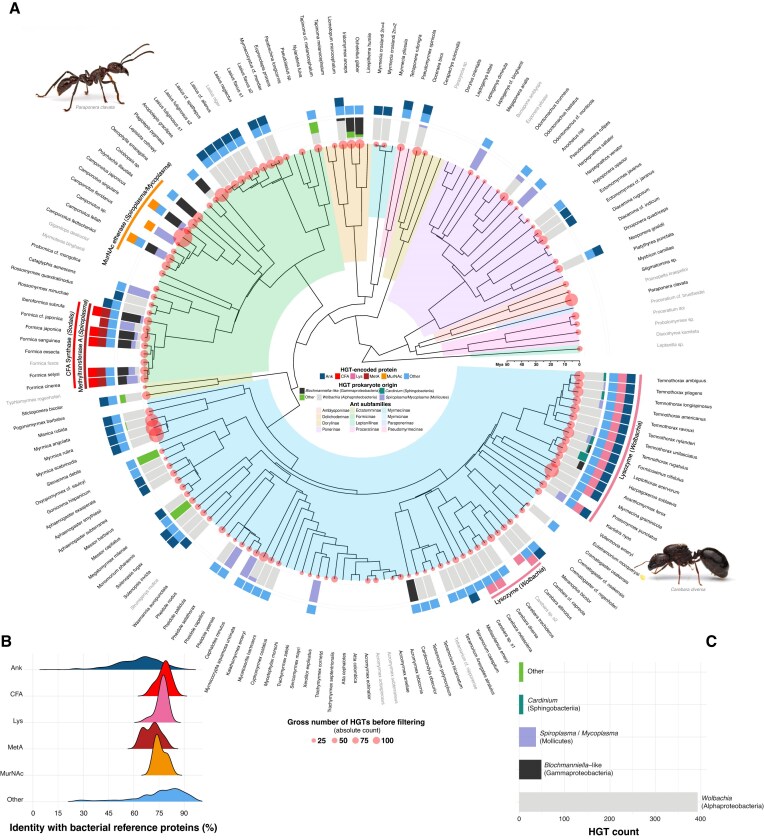
Phylogenetic distribution, prevalence, and origins of bacterial HGTs in ants. (A) Species phylogeny of 163 analyzed ant genomes [32] with presence/absence and inferred origins of bacteria-to-ant HGTs identified by an automated pipeline. Background colors in the phylogeny indicate ant subfamilies. Red points at branch tips denote the number of candidate HGT loci prior to manual curation (*n* = 1,148 loci; 7,348 putative HGT events). Stacked bar plots show the relative contribution of prokaryotic donors, with *Wolbachia* (light gray) most prevalent, followed by *Candidatus Blochmanniella*-like bacteria (black), *Spiroplasma/Mycoplasma* (purple), *Cardinium* (dark green), and other bacteria (light green). The outer ring indicates presence/absence of HGT-encoded proteins. Ankyrin repeat proteins (Ank, dark blue) are most abundant. Cyclopropane formic acid (CFA) synthases (red) and RNA methyltransferases (MetA, dark red) are restricted to Formicini, while Lysozymes (Lys, pink) occur in *Carebara, Temnothorax*, and related genera. *N*-acetyl-muramic acid etherases (MurNAc, orange) are restricted to Camponotini. Other proteins are highlighted in light blue (Table S1). Additional candidates, which were detected during in-depth analyses of clade-specific HGTs (CFA, Lys, MetA, MurNAc) are not highlighted in the Figure, but mentioned and described in the main text, as well as in Fig. 2 and in Tables S5–S7). Species with short-read stLFR genome assemblies are indicated in gray. (B) Percentage identity of HGT loci relative to inferred bacterial donors (CDS level). Conserved clade-specific HGTs (CFA, Lys, MetA, and MurNAc) show ∼75% identity (range ∼60–90%), whereas ANKs and other HGTs exhibit a broader range (20–100%). (C) Taxonomic distribution of inferred bacterial donors for 497 HGT loci. *Wolbachia* accounts for 79% (*n* = 393), followed by *Candidatus Blochmanniella*-like bacteria (*n* = 49), *Spiroplasma/Mycoplasma* (*n* = 37), *Cardinium* (*n* = 9), and other bacteria (*n* = 9, Table S1). Photo credits: *Paraponera clavata* ©Alex Wild; *Carebara diversa* ©Eduard Florin Niga.

To systematically identify HGT from bacteria to ants, we used a conservative approach, favoring specificity (accepting false negatives) over sensitivity (avoiding false positives). We screened 163 ant genomes from 12 subfamilies (Fig. 1A), namely Amblyoponinae (3), Dolichoderinae (6), Dorylinae (4), Ectatomminae (2), Formicinae (39), Leptanillinae (1), Myrmeciinae (3), Myrmicinae(77), Paraponerinae (1), Ponerinae (21), Proceratiinae (4), and Pseudomyrmecinae (2). Using a homology-based sliding window approach (2 kb genomic windows, 500 bp overlap) against curated bacterial and insect genome databases (see the “Methods” section), we identified 13,664 potential HGT candidate sequences across the 163 investigated ant genomes, which were used as a starting point for downstream analyses. After careful filtering and manual curation (see the “Methods” section), we identified 497 high-confidence HGT events (involving 1,053 protein-coding genes) of bacterial origin across 85 ant genomes (Fig. 1A, Table S1). The highest numbers of HGTs were found in three *Myrmica* species (*M. scabrinodis* (40 HGTs), *M. rubra* (38), and *M. angulata* (28)). We did not find any HGTs in the leaf-cutting ants *Atta* (*A. cephalotes, A. colombica*) or *Acromyrmex* (*A. ameliae, A. echinatior, A. lobicornis*), despite high-quality reference genomes and previously described prevalence of endosymbionts [35, 36]. We did not classify any HGT candidate as high-confidence in several lower-quality genome assemblies (species labeled in gray in Fig. 1), including the two most basal subfamilies of Leptanillinae and Proceratiinae, because long reads supporting the integration of the HGT in the host genome were not available in these cases. However, the number of candidate HGTs in these lower quality genomes before filtering was similar to the numbers in other more contiguous assemblies (Fig. 1A), suggesting that the prevalence of HGTs is similar across ant subfamilies.

We used PCR and Sanger Sequencing to confirm a subset of the computationally predicted HGT loci at the molecular level. Out of the 43 tested HGT candidate events, 36 could be confirmed by PCR and Sanger Sequencing, while results for seven remained inconclusive (Table S2). Additionally, we compared our list of identified HGTs with the few previously reported bacterial HGTs in ants [11, 33, 34] and found that several HGTs were recovered in our study (Table S3).

The 497 identified HGT loci contained coding sequences (CDS) for 1,053 bacterial proteins (Table S1). Among these, genes coding for ANK proteins were most abundant, identified in 45 ant genomes from eight subfamilies and with broadly distributed sequence identity percentages relative to their respective bacterial reference proteins (Fig. 1B). We further detected four clade-specific HGTs conserved across several closely related species: (i) Cyclopropane-Fatty-Acyl-Synthases (CFA) and (ii) Ribosomal RNA methyltransferases (MetA) in eight Formicini (Formicinae) species (the wood ants *Formica* and *Iberoformica*), (iii) Lysozymes (Lys) in 21 species belonging to two different clades in the Myrmicinae (*Temnothorax* acorn ants and *Carebara* marauder ants), and (iv) *N*-Acetyl-muramicacid-6-P-etherases (MurNAc) in eight Camponotini (Formicinae) species including carpenter ants (Fig. 1A, B). All clade-specific HGTs exhibited an average sequence identity of approximately 75% to their closest bacterial reference sequence, a pattern consistent either with adaptive divergence following HGT integration or with the possibility that the true bacterial donor has not yet been sequenced (Fig. 1B). We also identified seven cases of HGT shared between two or three distantly related species, of which two showed conserved synteny suggesting a single origin (Fig. S1, Table S4). Finally, we detected 61 HGTs unique to single species (Table S1).

CDS lengths of annotated HGT loci ranged from 150 to 10,000 bp, with 58 HGTs having lengths > 6,000 bp. Out of the 1,053 annotated CDS sequences, 384 were expressed (read counts > 100, see the “Methods” section for a detailed description of the available RNAseq data and their analysis), consistent with these genes carrying biological function. Gene ontology (GO) term enrichment analyses across all annotated HGTs highlighted enrichment in lipid biosynthesis, prokaryotic cell wall catabolism, bacterial cell wall degradation, methylation, and nucleotide metabolism (Fig. S2).

*Wolbachia* endosymbionts (Alphaproteobacteria) were the most frequent source of HGTs, accounting for 79% of the 497 identified loci in the ant genomes (Fig. 1A, C). Gammaproteobacteria (*Candidatus Blochmanniella* and related genera) contributed 10% (*n* = 49), while *Spiroplasma/Mycoplasma* (Mollicutes) were donors for 37 HGTs, followed by Sphingobacteriia (*n* = 9) and other, not further specified, bacteria (*n* = 9, Fig. 1C, Table S1). Ants of the subfamily Myrmicinae showed high prevalences of *Wolbachia*-derived HGTs, along with lineage-specific acquisitions of *Spiroplasma* and *Cardinium* (Sphingobacteriia) in *Temnothorax* and close relatives (Fig. 1A). The Formicinae and Dolichoderinae subfamilies exhibited a greater diversity of bacterial HGT donors, with Enterobacteria (e.g., *Sodalis, Yersinia*, or *Candidatus Blochmanniella-like* bacteria) contributing 25% across the Formicinae genomes and 51% across the Dolichoderinae genomes (Fig. 1A, Table S1).

### Widespread convergent HGTs of *Wolbachia* Ankyrin repeat genes

245 HGTs (49% of the 497 total) encoded one or multiple ankyrin repeat (ANK) proteins, distributed across 45 ant species from eight subfamilies (Fig. 1, Fig. S3). ANK HGT frequencies ranged from one (in twelve ant species) to 42 and 64 in *Myrmica scabrinodis* and *Myrmica rubra*, respectively. Out of the 418 annotated ANK proteins, 249 were expressed across 38 ant species from seven subfamilies. Notably, 80 of these 249 expressed ANKs were found in the genus *Myrmica*, a significant overrepresentation of bacterial ANK repeats in this genus (Fisher’s exact test, *P* < 0.0001, odds ratio = 7.31).

All ANK HGTs originated from *Wolbachia*. Using annotations from Uniprot, we identified 19 different *Wolbachia* strains as potential donors. The number of ANK proteins attributed to individual *Wolbachia* strains ranged from one to 132, though no consistent pattern was observed within *Wolbachia* species or across host ant species (Fig. 1, Fig. S3, Table S1).

Predicted ANK HGT proteins could be assigned to 21 different UniRef clusters, based on homology, varying in length (189 to 4751 amino acids) and domain architecture within and between ant species. Major ANK clusters recurred broadly across host ant species and subfamilies suggesting ancient origins without a discernible pattern (Fig. S3). ANK loci often consisted of many ANK genes in close proximity to one another, indicating secondary tandem duplications of *Wolbachia*–derived gene sets. The frequent expression of ANK HGTs seems incompatible with a purely neutral scenario. On the other hand, if ANK HGTs had straightforward adaptive roles (either for the ant or for *Wolbachia*), we could expect their expression to be positively correlated with sequence conservation relative to the (supposed) bacterial reference proteins. We did not find such a positive correlation (Pearson correlation coefficient *r* = −0.029, *P* = 0.55, Fig. S4), leaving the question of the adaptive function of these genes unanswered.

### Independent cases of ancient orthologous HGTs in different clades of ants

**Figure 2:**
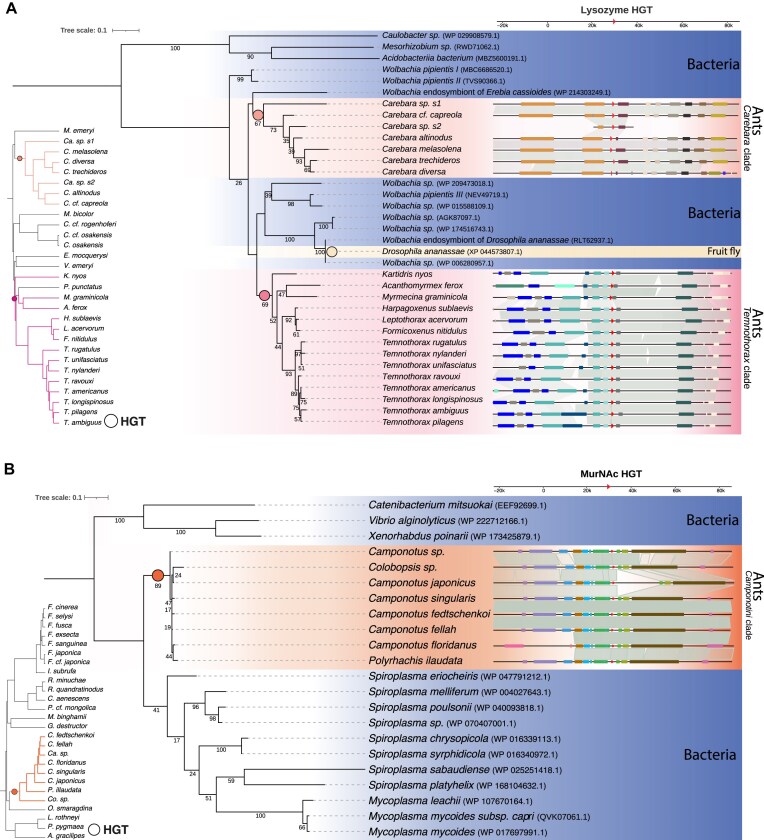
Representation of selected ancient orthologous HGTs. (A) Phylogeny and synteny of lysozyme (Lys) HGT loci in two Crematogastrini ant clades, *D. ananassae*, and putative bacterial donors. The phylogeny was inferred from HGT candidate protein sequences and their five best BLASTp hits from the NCBI non-redundant database and rooted on the bacterial lineage including *Caulobacter, Mesorhizobium*, and *Acidobacteriia*. Bootstrap values are shown at nodes (values <20 omitted). The lysozyme gene identified in *D. ananassae* was identified through a BLAST search and is included for comparison, but was not analyzed further. Synteny plots for the *Carebara* and *Temnothorax*-like clades show genomic neighborhoods surrounding HGT loci. Colored circles indicate putative independent HGT events, with orthologous genes shown in matching colors. The focal HGT candidate is marked by a red triangle. Homologous regions between genomes are indicated by gray connecting bars. The lysozyme HGT was not detected in the genome of *P. punctatus*, therefore this species is not included in the alignment of HGT sequences within this Crematogastrini clade. (B) Phylogenetic tree and synteny of MurNAc etherase genes horizontally transferred into the genomes of Camponotini ants and their putative bacterial donor lineages. The tree was inferred from the HGT candidate protein sequence of all respective taxa, including the five best BLASTp hits for each ant-derived HGT candidate obtained from the NCBI non-redundant database. The phylogeny was rooted on the bacterial branch leading to *Catenibacterium, Vibrio*, and *Xenorhabdus*. Bootstrap support values are indicated on the nodes, with values below 20 omitted for clarity. The synteny plot illustrates the genomic context surrounding the MurNAc HGT loci in Camponotini ants, with the orange-colored circle denoting the putative HGT event. Orthologous genes are shown in the same color, and HGT candidates are highlighted with red triangles similar to Fig. 2A. Gray bars indicate homologous genomic regions shared between species.

In-depth comparative analyses revealed four independent HGTs conserved as orthologs across different ant species (Fig. 1), all but one of which were expressed (Tables S5, S6, S7). These expressed HGTs code for (1) a bacterial lysozyme, derived from *Wolbachia* and acquired independently by the common ancestor of *Carebara* and the common ancestor of *Temnothorax* and *Kartidris* ants (Fig. 1, Fig. 2A, Fig. S5); (2) an *N*-Acetyl-Muramic-Acid-Etherase (MurNAc) originating from *Spiroplasma* in all Camponotini (Fig. 1, Fig. 2B); and (3) a Cyclopropane-Fatty-Acyl-Synthase (CFA Synthase) locus, derived from Enterobacteria and conserved in *Formica* and its sister genus *Iberoformica* (Fig. 1, Figs S6, S7). These HGTs showed conserved synteny across multiple species, consistent with purifying selection acting to preserve these regions.

Bacterial lysozyme HGTs were present in 21 ant species from two distinct Crematogastrini clades, one including several species of *Carebara* and the other including several species of *Temnothorax, Formicoxenus, Leptothorax, Harpagoxenus, Myrmecina, Acanthomyrmex*, and *Kartidris* (Fig. 1, Fig. 2A, Table S5). These lysozymes were all expressed with predicted transcripts containing a 5′-non-coding exon, consistent with the secondary emergence of gene regulatory structures (Fig. S5). Phylogenetic analyses characterized this HGT event in both ant clades as integrations of the *Wolbachia glycosyl hydrolase muramidase* lysozyme gene (GH25) into the ancestral ant genomes. Further, phylogenetic evidence revealed the insertions in the *Carebara* and *Temnothorax* clades to have evolved convergently (Fig. 2A), with a secondary loss in *Pristomyrmex punctatus* (Fig. 1, Fig. 2A). The evidence for convergent evolutionary acquisitions was supported by distinct patterns of highly conserved synteny in the genomic regions flanking the lysozyme HGTs in both the *Temnothorax* clade and *Carebara* (Fig. 2A). Based on divergence estimates of the different species, we estimate the convergent horizontal acquisitions of bacterial lysozymes in these ant clades to have occurred 29–39 MYA in the common ancestor of *Carebara* and ca. 51 MYA in the common ancestor of the *Temnothorax* clade, respectively.

We further identified a conserved MurNAc HGT (*murQ*) originating from Mollicutes bacteria in eight species of Camponotini (Fig. 1, Fig. 2B, Table S6), which was expressed in all eight species. Synteny and phylogenetic analyses confirmed the single ancestral HGT transfer into the ancestor of Camponotini 40–57 MYA (Fig. 2B).

Lastly, we identified a horizontally transferred *cfa* gene (encoding a CFA synthase) shared by eight species from the Formicini tribe (*Formica* and *Iberoformica*, Fig. 1A). Phylogenetic analyses revealed a likely origin from *Sodalis*-like enterobacteria and diversification into 87 *cfa* HGTs encoding 177 CDS sequences with 20 full-length expressed CFA genes (Table S7, Fig. S6). Each species had five to ten CFA synthases with lengths varying from short fragments to full-length CDS (Table S7). *Formica japonica* showed the highest number of full-length *cfa* genes (*n* = 5, all expressed), followed by *F. sanguinea* (*n* = 4, two expressed), and *F. cf. japonica* (*n* = 3, all expressed). *F. fusca, F. exsecta*, and *F. cinerea* all carried one complete and expressed *cfa* gene of 1,148 bp, while *Iberoformica subrufa*, the most basal of the eight species, had two complete and expressed CFA synthase sequences. Phylogenetic and syntenic relationships of the full-length CFA synthase HGTs suggested a single evolutionary origin ca. 33 MYA, followed by a complex evolutionary history with recurrent gene duplications, deletions, and/or translocations (Fig. S6, Fig. S7).

### Horizontally transferred genes in ants are functionally and taxonomically diverse

**Figure 3:**
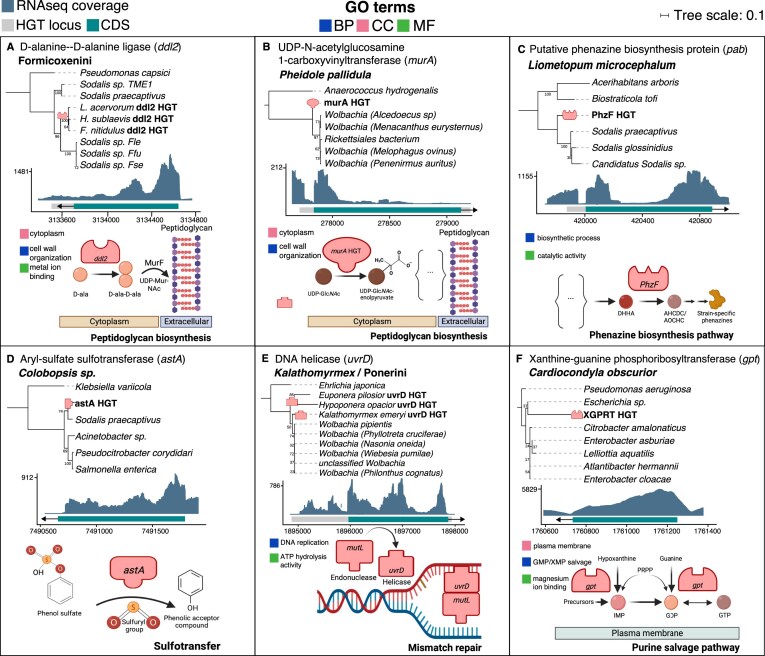
In-depth analysis of additional high-confidence HGTs in different ant species. Each presented HGT contains an expressed and full-length CDS, with a rooted phylogenetic gene tree constructed from the HGT protein sequence and the five best BLASTp hits identified in the NCBI non-redundant database, representing the most similar homologous bacterial proteins. RNAseq coverage is visualized for each HGT locus, with CDS regions shown in cyan. The focal HGT protein is highlighted in red within the phylogeny. Putative gene function is illustrated at the bottom of each panel. Associated GO enrichment terms, based on annotations from UniProt, are color-coded: Biological Process (BP; blue), Cellular Component (CC; pink), and Molecular Function (MF; green). (A) *D-alanine–D-alanine ligase* HGT locus in *L. acervorum, H. sublaevis*, and *F. nitidulus*, with *Sodalis* as the closest bacterial match. This gene is functionally associated with peptidoglycan biosynthesis, a key process in bacterial cell wall construction. (B) UDP-N-acetylglucosamine-1-carboxylvinyltransferase in *P. pallidula*. Closest bacterial match: *Wolbachia*. Associated with Peptidoglycan biosynthesis. (C) Phenazine biosynthesis protein in *L. microcephalum*. Closest bacterial match: *Sodalis*. Associated with the phenazine biosynthesis pathway. (D) Aryl-sulfate sulfotransferase in *Colobopsis* sp. Closest bacterial match: *Sodalis*. Associated with Sulfotransfer. (E) DNA helicase in Kalathomyrmex/Ponerini. Closest bacterial match: *Wolbachia*. Associated with DNA mismatch repair. (F) Xanthine-guanine phosphoribosyltransferase in *C. obscurior*. Closest bacterial match: *Escherichia* sp. Associated with the purine salvage way and recycling of nucleotides.

Among the 75 remaining HGTs that were neither ANK loci, fragmented, nor ancestrally conserved functional loci, we focused on six HGT candidates for further investigation which were all expressed and encoding full-length bacterial proteins of > 65% sequence identity (Table S8). Five out of these six could be confirmed by PCR (Table S2, for *Colobopsis* sp. no DNA was available). Two of these coded for proteins related to bacterial cell wall and membrane biosynthesis functions: An enterobacterial D-alanine–D-alanine ligase (*ddl2*) (Fig. 3A, Fig. S1, Fig. S8A) conserved in three Formicoxenini species (*Formicoxenus nitidulus, Harpagoxenus sublaevis, Leptothorax acervorum*), and *a Wolbachia*-derived UDP-*N*-acetyl-glucosamine-1-carboxyvinyltransferase (*murA*) in *Pheidole pallidula* (Fig. 3B, Fig. S8B). Note that RNAseq data indicate overlap of the *murA* CDS with an unannotated exon of gene *Ppal_g15224* on the reverse strand (Supplementary Fig. S8B), potentially causing an overestimate of the *murA* expression level. Additionally, four HGTs were associated with metabolic pathways (Fig. 3C–F, Fig. S8C–F): (i) a phenazine biosynthesis protein (PhzF) in *Liometopum microcephalum*, (ii) an Aryl-sulfate sulfotransferase (ASST) in *Colobopsis* sp., (iii) a DNA helicase (*uvrD*) involved in DNA mismatch repair in *Kalathomyrmex emeryi* and two Ponerinae (*Hypoponera opacior, Euponera pilosior*) and (iv) a Xanthine-guanine-phosphoribosyltransferase (XGPRT) in *C. obscurior*. Three of these were derived from *Sodalis*-like endosymbionts of the Enterobacteriaceae family (Fig. 3C, D, F) while the *uvrD* HGT in *K. emeryi, E. pilosio*r, and *H. opacior* originated from *Wolbachia* (Fig. 3E). For some of these HGTs (e.g., *PhzF* in *L. microcephalum* and *murA* in *P. pallidula*), gene expression patterns suggested the presence of regulatory 5′ non-coding exons (Fig. 3B, C).

We also identified HGTs of *uvrD* DNA helicases in two Ponerini species and in the myrmicine ant *K. emeryi* (Fig. 3E), which are likely to be three independent evolutionary HGT insertions into different genomic regions, according to synteny analyses (Fig. S1). Sequences of the ponerini *E. pilosior* and *H. opacior uvrD* CDS were more similar to one another than to sequences of *K. emeryi* and any of the *Wolbachia* strains (Fig. 3E). In general, this HGT showed over 30% divergence from the closest *Wolbachia* hit, suggesting that the true donor strain has not yet been identified but contributed the same HGTs to *E. pilosior* and *H. opacior*. The alternative interpretation of an ancient origin of the *uvrD* HGT in the common ancestor of *E. pilosior* and *H. opacior* would imply convergent losses in at least twelve other Ponerinae species (Fig. 1). Finally, the HGT in *C. obscurior*, coding for a Xanthine-guanine-phosphoribosyltransferase (XGPRT), a protein involved in the bacterial purine salvage pathway, showed a highly conserved CDS with high expression in the ant (Fig. 3F). This HGT has already been reported in a study by Klein et al. [34] and is suspected to be derived from the intracellular Enterobacteriaceae symbiont *Candidatus Westeberhardia cardiocondylae*.

### The horizontally acquired XGPRT gene has been co-opted into basic energy metabolism in the ant *C. obscurior*

**Figure 4:**
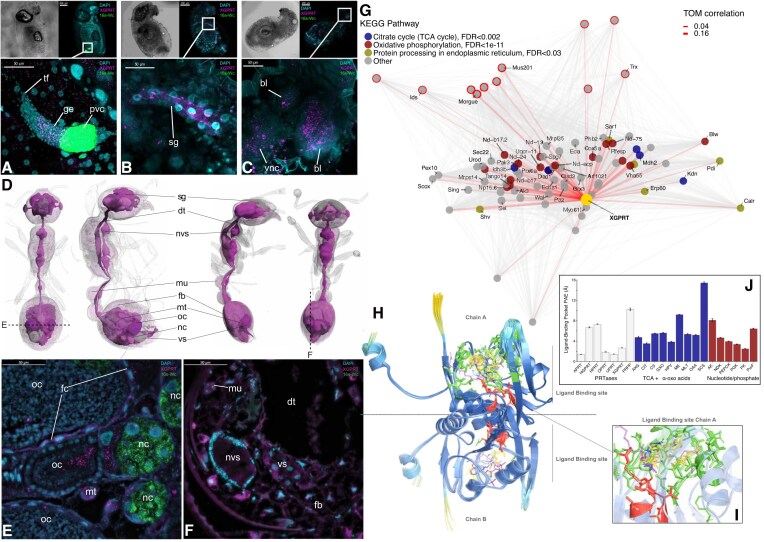
Functional characterization of the *C. obscurior* XGPRT HGT. (A–C) Localization of XGPRT expression by HCR-RNA-FISH in third instar larvae, detected in the apical germline of developing ovaries near *Westeberhardia*-infected pre-vitellogenic cysts (A), salivary gland epithelia (B), and brain and nervous tissue (C). Abbreviations: tf = terminal filament, ge = germarium, pvc = pre-vitellogenic cysts, sg = salivary gland, b = brain lobes, vnc = ventral nerve cord. (D) Overview of XGPRT expression (purple) in mature queens and workers. Anatomical reconstructions are based on microCT images (OKENT0105026, OKENT0105028); underlying HCR-RNA-FISH images are provided in Fig. S9. Abbreviations: dt = digestive tract, fb = fat body, mu = muscle, mt = malpighian tubules, nc = nurse cell, nvs = nervous system, oc—oocyte, sg = salivary gland, vs = venom system. (E, F) Cellular expression patterns in ovaries (E), and worker gaster tissues (F), including follicle cells, nurse cells, oocytes, venom system, fat body, digestive epithelia, muscle, and nervous system. (G) Gene co-expression network (*n* = 82) derived from transcriptomes of 36 third instar larvae. Nodes represent genes (XGPRT in yellow), arranged by principal components; edges indicate topological overlap (TOM), with XGPRT connections in red. Colored nodes in blue, red or dark yellow denote enriched KEGG pathways. Labels show assigned gene symbols. FDR values are Benjamini–Hochberg-adjusted *P*-values from KEGG pathway enrichment (hypergeometric test). (H, I) Structural analysis of AlphaFold3-predicted XGPRT dimers with diverse ligands. Structures are colored by prediction confidence (pLDDT), with binding pockets near a loop region (A39-L45). Residues within 5 Å of ligands are highlighted. (J) Predicted alignment error (PAE) for ligand-binding interactions across substrates. Lower PAE indicates higher confidence; values are grouped into high (<5 Å), moderate (5–10 Å), and low (>10 Å) confidence. Error bars show the standard error of the mean. Substrates are arranged by biochemical class: phosphoribosyltransferase (PRTase) substrates, TCA-cycle and α-oxo acid intermediates, and nucleotide/phosphate-transfer substrates.

*In situ* hybridization chain reaction assays in *C. obscurior* larvae revealed widespread expression of the horizontally acquired XGPRT gene, most prominently in developing ovaries (Fig. 4A), salivary glands (Fig. 4B), and brain and nervous tissue (Fig. 4C). Similarly, in adult queens and workers, we detected expression in various tissues and organs (Fig. 4D), including nervous tissue, muscles, fat body, malpighian tubes, salivary glands, venom glands, gut epithelia, bacteriomes and ovaries (Fig. 4E, F, Fig. S9). Within queen ovaries, expression was predominantly localized in the follicle cells of oocytes with comparatively weaker signals detected in oocytes as well as nurse cells containing *W. cardiocondylae* symbionts (Fig. S9E, G).

Gene co-expression and functional enrichment analyses of developmental transcriptomes from 28 individual third instar larvae [37] further assigned the XGPRT gene to a regulatory network of 83 genes enriched for basic cellular energy metabolism, including the citrate cycle and oxidative phosphorylation, and protein quality control functions (Fig. 4G, Table S9, Fig. S10).

To explore the potential ligand spectrum and enzymatic functions of the horizontally acquired XGPRT protein of *C. obscurior*, we used AlphaFold3, testing three classes of ligands: (1) canonical PRTase substrates, inferred from the conserved phosphoribosyltransferases (PRTase) domain (InterPro: IPR000836); (2) citric acid cycle intermediates and related α-oxo/hydroxy acids, motivated by co-expression with enzymes from these metabolic pathways; and (3) nucleotide/phosphate-transfer substrates, inferred from co-expression with proteins involved in oxidative phosphorylation. A detailed description of all reactions, substrates, and ligands is provided in the Methods and Supplementary Information.

We performed these analyses for the *C. obscurior* XGPRT and its seven closest prokaryotic homologs (Fig. 3F), modeling each in the multimeric states relevant for PRTases (dimers and tetramers; [38]. Across all ligands and homologs, we found that these XGPRTs display an unusually broad substrate spectrum, which is not typically associated with canonical XGPRT enzymes. The overall binding mode was remarkably consistent, and the core structure was highly conserved (Figs S11–14). Accordingly, XGPRTs showed high to moderate prediction confidence for ligand interactions across all three analyzed classes, with every ligand engaging the same binding pocket (Fig. 4H–J).

Structurally, the active-site entrance is formed by an N-terminal helix followed by a β–loop–α segment, with the loop region (A39–V–S–R–G–G–L) exhibiting all hallmarks of a noncanonical glycine-rich phosphate/carboxylate-binding loop (Fig. 4H, I). Comparable Gly/Ser/Arg-rich β-loop–α motifs occur in both classical Rossmann-fold dehydrogenases and P-loop NTPases [39–42]. Related SRGG/SRGGG-type motifs also function as ligand-binding loops in other systems, underscoring that this local chemistry of the XGPRTs is broadly suited for engaging diverse anionic groups such as phosphates and carboxylates [41].

Functionally, this β–loop–α binding surface provides a coherent explanation for how a prokaryotic XGPRT could become functionally integrated into central carbon metabolism and phosphate-transfer networks of an arthropod: The PRTase fold, with its adaptable β–loop–helix region, is inherently promiscuous. Its established ability to bind phosphorylated ligands such as PRPP creates a versatile binding pocket that can readily extend to TCA intermediates and other phosphorylated metabolites [43, 44].

## Discussion

In this study, we systematically investigated bacteria-to-ant horizontal gene transfers and identified 497 HGT loci encoding 1,053 genes in 85 ant species spanning eight subfamilies. Although our findings point to a rich functional and evolutionary diversity of HGTs, they likely represent a conservative estimate of the true prevalence of such transfers. The genomic and transcriptional signatures of the detected HGTs suggest functional integration and evolutionary significance, consistent with HGT-driven adaptive innovations in ant biology. Notably, the secondary acquisition of 5′ untranslated region (UTR) elements upstream of the start codon in several HGTs points to post-transfer fine-tuning by natural selection. Based on our data, we conclude that HGT has occurred repeatedly throughout ant evolution, with HGT-derived functional innovations most commonly linked to antibacterial defense and potential contributions to innate immunity (Fig. 3), as well as to metabolic enhancements and diversification (Fig. 4).

**Figure 5:**
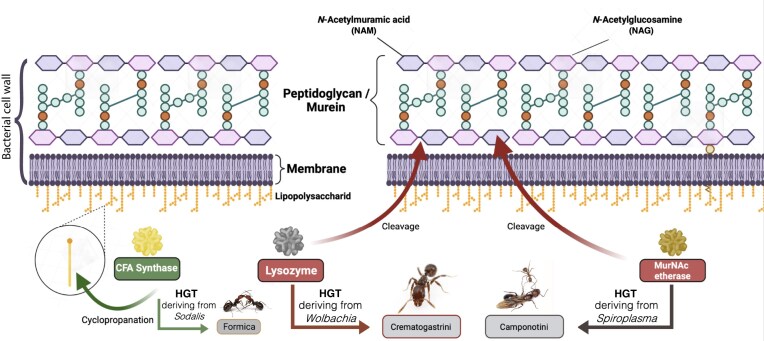
Schematic representation of conserved bacterial HGTs in ants with functions related to bacterial cell wall degradation. A bacterial cell wall consists of peptidoglycan/murein, accompanied by a membrane with associated lipopolysaccharides. The monosaccharide NAM occurs ubiquitously in the cell walls of gram-positive and gram-negative bacteria, forming the backbone of peptidoglycan together with *N*-acetylglucosamine (NAG). CFA Synthases are involved in the cyclopropanation of lipopolysaccharides, associated with bacterial stress responses and were detected as HGTs in *Formica* ants. Lysozymes cause a cleavage of peptidoglycan by acting on the bond between NAG and NAM (conserved in Crematogastrini ants), while *murQ* genes encode *N*-acetylmuramic acid 6-phosphate etherases (MurNAc etherases), which are bacteria-specific enzymes that can act upon NAM itself (conserved in Camponotini). Photo credits: *Formica obscuripes* ©Alex Wild; *Temnothorax americanus* ©Alex Wild; *Camponotus nicobarensis* ©Alex Wild.

Importantly, direct donor-recipient contact is a necessary condition for bacterial HGTs to the host germline. This implies that hosts with intracellular and vertically transmitted symbionts are most likely to experience symbiont-mediated HGT. This explains in turn why we expect bacterial HGTs to be variably prevalent in insects where vertically transmitted symbionts are common, but largely absent in vertebrates [27, 28]. In accordance, most bacteria-to-insect HGTs (also in the present study) involve *Wolbachia* (Fig. 1A, C), the most widely distributed maternally inherited intracellular symbiont of insects [45]. This finding aligns with previous studies showing that *Wolbachia* sequences of considerable length have been transferred to the nuclear genome of solitary insect hosts [11, 33, 46, 47]. The *Drosophila ananassae* genome even integrated an entire genomic copy of its *Wolbachia* symbiont in its own genome [46, 47]. Bacteriophages such as the temperate phage *WO* can mediate *Wolbachia*–derived HGTs, potentially enabling incorporation of genetic material from different *Wolbachia* strains in the same host genome [48]. Apart from *Wolbachia*, the intimate relationships of *Candidatus Blochmanniella* and *Blochmanniella*-like intracellular endosymbionts with e.g., *Camponotus, Plagiolepis, Formica*, and *Cardiocondyla* ants also provided opportunities for HGT. These have been documented in isolated ant lineages previously [49, 50], but are now shown to likely characterize entire Formicinae clades. Such HGTs from long-term coevolved endosymbiont lineages to their ant hosts (e.g., *Candidatus Blochmanniella-Camponotus*, approx. 80 million years) may play a critical role in reinforcing the functional relationship between hosts and these mutualists, thereby promoting sustained cooperation over evolutionary timescales. We also discovered HGTs from *Sodalis*-like endosymbionts in the Formicoxenini and the genus *Liometopum* (Fig. 5 A, C), despite such endosymbionts not occurring in extant populations of these ants, suggesting they constitute remnants of past symbioses or relied on other transmission routes.

While many HGTs are likely non-functional and will quickly be lost or degraded by mutation over evolutionary time, a subset of HGTs of bacterial origin can be co-opted for diverse roles in insect genomes and become fixed and conserved by natural selection [51–53]. Examples of such co-opted HGTs include genes contributing to pigmentation, modulation of courtship behavior, degradation of plant- or bacterial cell walls, enhancement of immune defenses, and improved detoxification capacities (reviewed in Husnik and McCutcheon [3] and Liu et al. [26]). In our study, HGTs often included genes involved in metabolic and cell-wall related processes in bacteria. For example, *uvrD* (a DNA helicase, Fig. 3E) and XGPRT (Fig. 3F) are well characterized in bacteria, but their function following HGT into the genome of ants remains unexplored.

Our in-depth functional analysis of the horizontally transferred XGPRT in *C. obscurior* revealed its association with fundamental metabolic pathways, specifically the citric acid cycle and oxidative phosphorylation (Fig. 4). The substrate promiscuity of XGPRT proteins provides a mechanistic explanation for how a single metabolic enzyme, conserved across bacterial lineages, can become functionally integrated into distinct metabolic pathways following horizontal transfer into a eukaryotic host. This biochemical flexibility may facilitate the rapid co-option of bacterial proteins into preexisting eukaryotic metabolic networks, not only in this but likely also in a number of other cases of bacteria to prokaryotic HGT [54, 55]. The horizontally transferred XGPRT is equipped with an inherently phosphate-competent catalytic scaffold, with the A39–V–S–R–G–G–L loop constituting a promiscuous docking pad enabling interaction with nucleotide salvage, the citric acid cycle, and phosphorylation pathways. By allowing for such an unusually broad substrate spectrum, this particular protein structure thus likely underlies the adaptive co-option of the acquired XGPRT into the metabolism of *Cardiocondyla* ants.

The widespread expression of the XGPRT across various ant tissues aligns well with its putative role in basic energy metabolism. This pattern becomes particularly striking in metabolically demanding secretory and reproductive tissues (e.g., the larval salivary glands, worker venom glands, and queen ovaries). Here, XGPRT expression was particularly strong indicating the capacity to match expression to energetic demands of different tissues and physiological contexts.

More comprehensive functional studies will be necessary to resolve the specific metabolic contributions of the *C. obscurior* XGPRT protein and establish causal relationships with particular physiological states. However, such investigations remain technically challenging for most ants, as reverse genetic approaches (CRISPR, RNAi) have only been established in very few model species of ants [56–58] and never as routine techniques. However, as our study demonstrates, HCR *in situ* hybridization, co-expression experiments, and structural predictions are valuable, accessible techniques that can help home in on the functional significance of horizontally acquired genes in non-model species. Similar investments in targeted functional studies are feasible across most ant species and will be necessary to further resolve the functional role of horizontally acquired genes in other ant lineages.

Overall, we find a number of ant HGTs most likely functionally associated with defenses against pathogens, often via bacterial cell-wall degradation (Fig. 5). Key examples are the clade-specific Lysozymes in several Myrmicinae species and the MurNAc etherases in Camponotini ants (Fig. 2). Lysozymes can serve as a protection from pathogens by peptidoglycan cleavage between *N*-Acetylglucosamine (NAG) and *N*-Acetylmuramic acid (NAM), while MurNAc etherases act in similar ways directly on NAM [59, 60]. These HGTs might provide antibacterial defense systems to the ants, killing pathogenic bacteria by cell wall degradation (Fig. 5). Notably, bacterial lysozyme genes have undergone independent horizontal transfers into a wide range of organisms, including viruses, fungi, archaea, plants [16], bivalves [61, 62], and solitary insects [16, 23, 63], where they have been functionally integrated. In some cases, such as in plants and archaea, these transfers have given rise to antibiotic GH25 muramidases, underscoring their potential adaptive significance [16]. Disease defenses are well documented to be a pervasive threat to ant colonies, that has maintained selection for multilayer recognition and immune defense mechanisms, so the HGTs discovered here add to a much broader spectrum of individual and social immune strategies [64].

In contrast to lysozymes, the HGT of *murQ* genes has not been reported previously and is potentially unique to the Camponotini ants. HGT-encoded *murQ* enzymes can convert *N*-acetylmuramic acid-phosphate to *N*-acetylglucosamine-phosphate by cleavage of the lactyl residue, which can then be further degraded, used in glycolysis, or directed into peptidoglycan *de novo* synthesis and recycling [59, 65, 66]. The *murQ* HGT has a strong adaptive potential in enhancing the ants’ immune defense by using such peptidoglycan-degrading enzymes to kill bacterial pathogens, while leaving an endosymbiotic relationship with cell-wall deficient *Spiroplasma*, the presumable HGT donor, unaffected [67].

CFA synthases, such as those acquired by two sister lineages of Formicini, catalyze the cyclopropanation of unsaturated fatty acids of bacterial membranes, which has been associated with adaptive stress responses to changes in pH, temperature, and salinity in bacteria (Fig. 5) [68–72]. CFA synthases have previously been identified in various eukaryotic lineages, including plants [73], fungi [74], and *Leishmania* parasites [71, 75, 76]. Evidence suggests that in several cases, these genes were horizontally acquired from bacteria, similar to what we observe for CFA synthases in the present study. We found that most Formicini species have several expressed, likely functional *cfa* gene copies and that full-length CFA synthases retained conserved synteny. This suggests that CFA synthases emerged from a single ancient HGT to the common ancestor of *Formica* and *Iberoformica* ca. 33 MYA [77] with secondary diversification by gene duplications and rearrangements (Fig. S3), coinciding with the adaptive radiation of the genus *Formica*. Finally, the *ddl2* HGT in three Formicoxenini species and the *murA* gene in *P. pallidula* potentially convey antibacterial functions as well, as both enzymes are involved in peptidoglycan anabolism and catabolism [78–80].

In contrast to these genes likely involved in immune function, the most prevalent HGTs across ant genomes encode ANK-domain proteins acquired from *Wolbachia*, suggesting a distinct evolutionary origin and functional significance. These ANK-domain proteins occur across 45 species from eight subfamilies and often in high copy number (Fig. 1A, C). ANKs consist of relatively short, tandem repeat motifs that fold into structures mediating molecular recognition via protein-protein interactions [81–84]. They are involved in a diverse set of functional host-symbiont interactions and may be employed by symbionts to mimic or manipulate host functions following infection of eukaryotic cells [85–88]. The general prevalence of ANK HGTs in insects suggests that they may continue to serve manipulative *Wolbachia* interests. However, it has been notoriously difficult to document that *Wolbachia* symbionts express reproductively parasitic phenotypes in ants [89], so their ANK HGTs might also extend finetuning of mutualistic functions.

## Conclusions

Ants are one of the most diverse insect families worldwide. Their social family structures, colony sizes, and ecological niches vary enormously, and our study indicates that regular HGTs from bacterial endosymbionts may have allowed a number of ant lineages to further finetune their fit to particular ecological niches. This perspective would be consistent with inferred HGT-mediated adaptations in other eukaryotes [90, 91]. However, it is important to emphasize that most HGTs become subject to genomic degradation and pseudogenization [92]. Nevertheless, our study recovered numerous convincing cases of HGTs that likely mediate adaptive responses to environmental challenge, often with strong signatures of evolutionary conservation and secondary elaboration (e.g., the incorporation of introns and UTRs) over time. In that sense, the fate of HGT events is the same as of any other mutation in the genome—they are most likely to persist and not degrade when they convey an adaptive benefit. Only after such positive maintenance directly following HGT can secondary elaborations become part of broader gene regulatory networks that mediate complex phenotypic traits, consistent with conjectures brought forward in the past [11]. The results reported here should encourage further research, both to extend coverage across the ants (as the 163 Global Ant Genomics Alliance [GAGA]-generated genomes represent just over 1% of the total number of described ant species) and to probe HGT functionality in greater detail at the level of specific tribes or genera.

## Methods

### Taxon sampling

The vast majority of all investigated ant genomes were sampled, sequenced, and annotated by the GAGA [32, 93]. Information about the collection, sequencing, assembly, and annotation methods, as well as detailed sample descriptions can be obtained from [32]. Briefly, this dataset included 145 genomes sequenced and assembled by GAGA, as well as 18 previously published genomes re-annotated with the same gene annotation pipeline (Table S10). Our total dataset thus contained 163 species distributed across 99 genera (i.e., 29% of the 347 known genera), from 12 out of the 17 extant ant subfamilies. 143/163 genomes were PacBio-sequenced and assembled and had sufficient contiguity to reliably identify HGT candidates. For 15 of the 143 PacBio sequenced species, it was possible to obtain chromosome-resolution genomes using Hi-C sequencing (Table S10). The remaining 20 of the 163 ant genomes were assembled from short-read stLFR (single-tube long fragment read) data and exhibited low contiguity (light gray species names in Fig. 1A). Although initial candidate HGTs were identified in these assemblies, none passed manual curation due to insufficient contiguity to support any predictions meeting our stringent filtering criteria. Comprehensive details on genome assemblies, gene annotation procedures, and assessments of assembly completeness are provided in [32].

### Detection, validation, and quality assessment of HGT candidates

All 163 ant genomes were first screened for contaminating bacterial scaffolds (described in detail in [32]) and subsequently for candidate regions of horizontal gene transfer from bacterial donors using a homology-based approach. For this, all sequenced ant genomes were divided into sliding windows of 2 kb (with 500 bp overlap) and searched against a curated prokaryotic and two insect genome databases (one including four ant genomes and one without ant genomes) with *mmseqs2* (release_12–113e3, with “–start-sens 1 –sens-steps 2 -s 7 –search-type 3” followed by “mmseqs convertalis” [94]) to quantify similarity to published prokaryotic or insect genomic sequences. The different databases contained (i) 1,908 complete bacterial genome sequences from PATRIC (Table S11) or (ii) 43 (including four ant genome assemblies) or (iii) 39 (without ant genomes) “Chromosome”-level or “Complete Genome”-level insect genome assemblies from NCBI, which were further filtered by *blobtools2* (using “Insecta”) to remove putative contaminations with bacterial sequences in these reference assemblies (Table S12). For each sliding window of each genome, the best scoring hit from each database was sorted by evalue (-k 7,7 g) and bitscore (-k 8,8gr). Bacterial and eukaryotic rRNAs in the ant genomes were annotated with *barrnap* [95] and overlapping sliding windows identified with *bedtools intersect* v2.28.0 [96]. We used *infoseq* (emboss 6.6.0, with “-nocolumn -delimiter “t” -auto -only -name -length -pgc”) to calculate GC content and *profileComplexSeq.pl* to calculate different measures of sequence complexity (e.g., entropy, Trifnov’s complexity, see below) in each sliding window. We next mapped the available raw genomic reads (with “-ax map-pb” for PacBio long-reads or “-ax sr” for short-reads) against the corresponding assembled genome using *minimap2* (version 2.17r941, [97]). After sorting aligned reads with *samtools sort* v1.9 [98], we used *bedtools coverage* v2.28.0 [96] to calculate coverage in each sliding window.

We then filtered all sliding windows to identify HGT candidate regions based on the following criteria: (1) at least one high-scoring segment pair (HSP) against the bacterial database has an e-value of <1e-5, (2) the bitscore difference between the best hit against the bacterial database and the insect database (either with or without ants) is >100. (3) The HSP against the bacterial database is longer than 100 bp. Sliding windows retained after filtering that were less than 500 bp apart were merged into HGT candidate loci (defined as one common “HGT event”) for further analyses, resulting in 13,664 predicted HGT candidate loci.

To evaluate the quality of the 13,664 candidates in downstream analyses, detailed overview plots with multiple HGT-quality parameters were produced for each genome and each predicted candidate locus (Figs. S15–S18, giving examples of these produced overview plots of selected HGT candidates for quality evaluation). The overview plots for each HGT candidate contained the following information. (1) log10-scaled bitscores of the highest scoring hits against the bacterial and insect (with or without ants) databases for all sliding windows from −200 kb to +200 kb surrounding the candidate HGT locus, (2) the relative coverage (log2-scaled) of each sliding window calculated by dividing the number of reads mapping to each sliding window by the average genome-wide coverage, (3) the alignment positions of all reads overlapping the candidate HGT locus, (4) the position of the best hit against the prokaryotic database and against the SwissProt database, together with corresponding *e*-value and organism.

To assess appropriate filtering cutoffs for the whole dataset, predicted HGTs from seven randomly selected GAGA genomes were evaluated manually using the aforementioned overview plots, and parameter distributions were plotted for all 13,664 HGT candidate sequences (Fig. S19). Evaluated parameters for HGT detection were the following: (a) BitDiffSum (i.e., the differences in bitscores retrieved from homology searches against the different databases), (b) candidate length, (c) ce (Entropy), (d) ct4 (Trifnov’s complexity with order 4), (e) GC content, (f) locus length, (g) number of reads overlapping the start of the HGT sequence, and (h) number of reads overlapping the end of the HGT sequence. After evaluating these criteria for randomly selected candidates, general filtering criteria were defined as follows: *e*-value > 1e-25 against the prokaryotic database, ct4 > 0.25, ce > 1.5, BitDiffSum > 150, and candidate length > 100 bp for all candidate loci to yield an unbiased selection of high quality HGT candidates. The filtering steps to remove false-positives were performed in R (version 4.1.2), using the packages *data.table, dplyr, tidyr, tidyverse*, and *stringr*. All remaining HGT candidate loci (*n* = 1,149, Table S13) were then subject to further manual curation by inspecting alignments of raw sequencing data against the predicted candidate loci and used as input for a prokaryotic gene annotation.

#### PCR and Sanger sequencing of HGTs

Genomic DNA of 25 available GAGA samples was extracted using a Chelex protocol to verify incorporation of detected HGTs into their respective ant genomes. PCR Primers were designed with a length of 18–22 bp, Tm between 58 and 62°C, and high target specificity (i.e., no off-target binding sites) for all possible HGT candidates. Primer pairs were also required to span the expected amplicon as a fragment of the predicted HGT CDS in combination with the ant DNA in both up- and downstream directions of the HGT (Table S2). The amplification of PCR products was verified using agarose gel electrophoresis. Correctly amplified PCR products matching expected size were then sequenced using Sanger sequencing technology after which chromatograms were re-aligned to the reference genome to confirm HGT presence within the ant genome.

#### Evaluation of border regions between ant DNA and bacterial HGT

Border regions between predicted HGT regions and adjacent host DNA were closely examined to identify missassemblies and chimeric bacterial-ant scaffolds. Using alignments of the previously mapped genomic raw reads (see above), reads overlapping each predicted HGT region were extracted and quantified using *bedtools intersect*. Specifically, read counts at the 5′ and 3′ boundaries of each candidate region were used as an additional filtering criterion. Candidates with fewer than two reads overlapping the boundary between ant and bacterial sequence were classified as likely missassemblies and excluded from further analysis. To accurately define HGT boundaries and support read mapping, we calculated the average read length distribution across all GAGA genomes, separately for stLFR and PacBio-based assemblies. Read support was then assessed at three positions: the 5′ boundary, the 3′ boundary, and across the entire candidate region (including a flanking extension of 1,000 bp for PacBio and 25 bp for stLFR assemblies on both ends). Following filtering, adjacent HGT regions were either merged or split based on manual inspection, guided by continuous homology to bacterial sequences determined via BLAST bit scores. Ultimately, all HGT regions were resolved into discrete, high-confidence candidate sequences.

Our strict filtering criteria led us to exclude the low-contiguity stLFR-based genome assemblies at this point of the analysis as inspection of mapped short-read data did not allow for conclusive discrimination between assembly artifacts and properly integrated HGT events.

### Prokaryotic gene annotation and functional analyses

Protein-coding and non-coding genes were annotated for all high-quality HGT candidates, using a combination of Prodigal [99], Kraken2, and DFAST [100]. All high-quality HGT candidates CDS sequences were then searched against NR and NT databases downloaded from NCBI, bacterial protein sequences included in UniProt90, and TIGRFAM and COG databases. For DFAST we required a minimum-length of 100 bp for all bacterial reference sequences while including the *–metagenome* option for incomplete genomes.

#### Examining gene completeness and identification of fragmented HGTs

To investigate gene completeness and identify fragmented, putatively non-functional HGTs, we extracted start and stop codons of predicted coding gene sequences (CDS) from all resulting DFAST files using *SeqKit* [101]. Accordingly, parameters reporting query coverage (q_cov), subject coverage of the bacterial reference (s_cov), and e-value were examined to identify cases of incomplete or fragmented HGTs. By default, query sequences with a subject coverage < 75% were marked as partial hits by DFAST. We additionally used Geneious Prime [102] to visually inspect open reading frames (ORFs) and completeness of selected HGT candidates. Our analyses concluded that several HGT regions had been too narrowly defined, rendering many CDS of HGTs truncated. To complete such fragmental and undersized HGT candidates resulting from our too conservative filtering, we extended all HGT loci by 1000 bp at the 5′ and 3′ boundary and annotated again with DFAST. All reannotated sequences were then intersected with the originally predicted CDS using *bedtools* [96] to make sure that we only extended previously obtained loci. A summary covering both the original annotation and the reannotation is provided in Table S1, which covers all identified HGTs. Additionally, we integrated information from UniProt (retrieved with *UniProtR* [103]) to obtain sequences from the closest bacterial homolog from UniProt90. This included GO terms, protein names, and predicted bacterial reference taxa (Fig. S2, Table S1).

#### Gene expression analysis of HGTs

We used RNAseq data available for 130 of the 163 studied ant species to assess gene expression of the HGT loci. RNAseq data was made available and collected by the GAGA project. Information about available RNAseq data for each investigated species, including the sampling of different ant castes and developmental stages, can be obtained from Table S1A in Vizueta et al. (2025). First, paired-end short-read RNAseq data were mapped to the corresponding genomic regions using STAR v2.7.2b [104] with stringent alignment parameters only allowing > 99% identity and > 90% alignment lengths (–outFilterMismatchNoverReadLmax 0.01 –outFilterScoreMinOverLread 0.9 –outFilterMatchNminOverLread 0.9). As reads were mapped exclusively to candidate HGT regions rather than full genomes, we only report absolute read counts, using a threshold of >100 raw mapped reads as a conservative criterion to indicate robust transcriptional evidence of candidate HGT loci. For each ant species, we merged mapped reads from different samples using *samtools* [98] and retained only uniquely mapped reads overlapping with predicted HGT genes. We finally estimated overall gene expression for every candidate HGT and reported them as raw read counts (Table S1).

### Comparative genomic analysis of selected HGT candidates

We analyzed in detail all remaining expressed HGTs (read count > 100), which had: (i) < 80% coverage of the annotated Uniprot hit (to reduce the possibility of fragmented or wrongly annotated HGTs, while still considering different evolutionary trajectories), (ii) at least 65% identity with the identified bacterial donor sequence to ensure bacterial origin, and (iii) a complete ORF verified by the NCBI ORF finder. For these HGTs, we manually verified completeness of each CDS by conducting BLAST searches, comparing ORFs, using the GAGA annotations [32] and incorporating RNAseq data.

Gene models of these HGTs were manually refined in Geneious, using transcripts obtained with *StringTie* (default settings, [105]) as guides. In cases where several exon-intron structures were predicted, we used parsimony to manually select a single representative model based on the RNAseq data. Synteny analyses were conducted for all candidate HGTs occurring in narrow phylogenetic clades of ants to evaluate the conservation of the HGT regions. For this purpose, every HGT locus was extended by 40 kb on each side after which all ant genes and protein sequences within this flanking region were extracted. *Minimap2* [97] was then used to conduct an all-vs-all alignment after which OrthoFinder [106] was used to determine orthogroups across species. The extent of synteny was plotted with the R package *gggenomes* [107]. Finally, the candidate HGT sequences were blasted against all GAGA ant genomes to uncover additional HGT events that might previously have been excluded due to our strict filtering criteria (Tables S5–S7). Using identified clade-specific HGT sequences as queries, we conducted a local blast against all GAGA genomes to uncover potential additional HGT events that were previously excluded because of our strict filtering criteria. The resulting blast hits were then again intersected with all HGT loci initially predicted by the automatic pipeline using *bedtools* [96], which showed that these additional HGT events had indeed been identified as candidate HGTs by the automated HGT finder pipeline, confirming that no HGT event was missed by that pipeline and that we may have filtered candidate HGTs that were real in our aim to avoid false positives. We extracted the FASTA sequences for all resulting intersected HGT candidates and ran DFAST again to annotate them. We also obtained gene expression and synteny data again for all of these selected clade-specific HGTs to complete the in-depth analyses.

Ultimately, we performed phylogenetic analyses based on protein sequence data to infer the putative evolutionary origins of selected HGT events. HGT protein sequences were annotated using DFAST [100], and the five most similar homologs for each candidate were retrieved via BLASTp against the NCBI non-redundant (nr) protein database. Multiple sequence alignments were performed using MAFFT with default settings [108]. Phylogenetic trees were then constructed using maximum likelihood inference in IQ-TREE2 [109], with node support assessed through 100 bootstrap replicates. Substitution model selection was performed automatically using ModelFinder, which is integrated within IQ-TREE and selects the best-fitting model based on statistical criteria such as the Bayesian information criterion. Phylogenetic trees were visualized and annotated using iTOL v4 [110] and evaluated for potential rooting ambiguity. Specifically, we assessed whether bacterial and eukaryotic sequences formed distinct monophyletic clades, following a strategy similar to Irwin et al. (2021). Trees were rooted on branches leading to *Caulobacter* sp. and *Mesorhizobium* sp. (Fig. 2A), as well as on branches leading to *Catenibacterium mitsuokai, Vibrio alginolyticus*, and *Xenorhabdus poinarii* (Fig. 2B). Detailed phylogenetic information for clade-specific HGTs—such as lysozymes, MurNAc etherases, and CFA synthases—as well as other HGT candidates, is provided in the supplementary materials, including synteny assessments and gene expression summaries (Tables S4–S8).

### XGPRT gene co-expression analysis

We used published RNAseq of 28 third instar larvae of *C. obscurior* [37] to identify the gene co-expression network comprising the horizontally acquired XGPRT gene in this species. Raw RNA sequencing reads were trimmed using trimgalore v0.6.10. Using STAR 2.7.11b [104], trimmed reads were mapped to the *C. obscurior* host (GCF_019399895.1, PRJNA1202182, [111]) and *Westeberhardia* endosymbiont genomes (GCF_001242845.1, PRJEB8217, [34]. We used BLASTn to identify and subsequently manually annotate the XGPRT HGT locus missing in the *C. obscurior* RefSeq annotation. The XGPRT HGT gene lies on the reverse strand on linkage group LG25 (NC_091888.1:913342–914012), with the CDS open reading frame ranging from bases 913875 to 913390.

For gene expression analyses, read counts generated with featureCounts v2.0.6 [112] were normalized and transformed to log2 counts per million. We used Bonferroni-corrected Pearson correlation coefficients to identify 82 *C. obscurior* genes significantly co-expressed with the XGPRT gene (Table S14) across samples. No *Westeberhardia* gene nor overall *Westeberhardia activity* showed significant co-expression (Fig. S20). Weighted gene co-expression network analysis was performed using the WGCNA package [113] in R. The adjacency network was built with a softPower of 13 and type = unassigned. We used *TOMsimilarity()* from the WGCNA package to calculate the topological overlap matrix (TOM) for all co-expressed genes. KEGG pathway enrichment was assessed using the *enrichKEGG()* function from the *clusterProfiler* package in R, with pvalueCutoff = 0.05, pAdjustMethod = “BH,” qvalueCutoff = 0.2, minGSSize = 0, maxGSSize = 500).

### Animal husbandry

Colonies of *C. obscurior* were kept in a climate chamber with a 12 h/12 h day/night rhythm at 26°C/22°C, respectively, and a constant humidity of 75%. The ants were fed with honey and cockroach (*Blaptica dubia*) three times a week. Water was provided *ad libitum*.

### Hybridization chain reaction RNA fluorescence *in-situ* Hybridization (HCR-RNA-FISH)

HCR-RNA-FISH was performed with gene-specific HCR^TM^ HiFi probes targeting mRNA of the horizontally acquired XGPRT gene from *C. obscurior* and targeting the *W. cardiocondylae* 16S rRNA, respectively, using the HCR^TM^ Gold RNA-FISH kit (Molecular Instruments). The amplifier sets X1-647 and X7-514 were used for XGPRT and *W. cardiocondylae* 16S rRNA, respectively.

### HCR-RNA-FISH with whole mount larvae

*Cardiocondyla obscurior* 3^rd^ instar queen- as well as worker-destined larvae were sampled into glass vials and fixed in 37% formaldehyde/2x PBS/heptane (1:1:2) for 2 h. Samples were washed with methanol, transferred to 1.5 ml Eppendorf tubes (Eppendorf), and washed again with methanol (30 min incubation). Larvae were rehydrated through a graded series of methanol-to-PBST exchanges (25%, 50%, 75%, 2×100%), with 10 min incubation each time. Probe hybridization and amplification were performed according to the manufacturer’s protocol (Molecular Instruments), with two modifications. For one, we performed two additional Saline sodium citrate and Tween (SSCT) washes for 5 min each after performing the standard HCR™ HiFi Probe Wash Buffer washes. Later, instead of using the HCR™ Gold Amplifier Wash Buffer we washed with SSCT, twice for 5 min and once for 30 min (adapted from [114]). Afterwards the SSCT was replaced with 1x PBS for 10 min and was then counterstained with 1 µl DAPI (1 µg/ml) in 1x PBS for 3 h in the dark at RT. The samples were washed with 1x PBS twice and 50/50 glycerol/PBS once for 10 min each. This was replaced with 70/30 glycerol/PBS. Whole larvae were mounted on slides using a drop of 70/30 glycerol/PBS and gene frames (Thermo Fisher Scientific). To assess signal specificity in the absence of a dedicated negative control probe, we evaluated background fluorescence in parallel HCR-FISH experiments using the same fluorophore (Alexa Fluor 647), revealing only minimal and diffuse nonspecific signal (Supplementary Fig. S21). Images were captured on an inverted DMi8 Stellaris (Leica Microsystems) using the Leica Application Suite X software.

### Micro-CT analysis

Micro-CT scans from *C. obscurior* queens (OKENT0105026) and workers (OKENT0105028) were obtained from Antscan built on the open-source online platform Biomedisa [115]. The reconstructions were analyzed using Dragonfly 2022.2 for Windows (Comet Technologies Canada Inc.).

### HCR-RNA-FISH with microtome sections

Specimens representing adult queens and workers were fixed in 80% *tert*-butanol containing 4% PFA for 24 h, with two biological replicates per category. The samples were pre-embedded in 1% agar and subsequently washed four times in 80% *tert*-butanol for 10 min each. Dehydration was carried out by incubating the samples in increasing concentrations of *tert*-butanol (90%, 96%, 3×100%) followed by isopropanol (3×100%) for 2 h each. The samples were infiltrated twice with paraffin at 60°C (2 h and 12 h, respectively) and then embedded in paraffin. Sagittal and coronal semi-thin sections (5 µm) were prepared using a Leica RM2245 rotation microtome with disposable blades. Following deparaffination and post-fixation in 4% PFA for 20 min, sections were digested with pepsin (0.4% in 0.9% NaCl, pH 1.5) for 15 min at 37°C. All steps were performed according to the manufacturer’s protocol, with the modification that after amplification and prior to the final washing, *C. obscurior* nuclei were counterstained with 1 µg/ml DAPI in 5x SSCT (100 µ per slide) for 1 h in a dark, humidified chamber at room temperature. After washing, the sections were mounted under high-precision coverslips using ProLong Diamond antifade mounting medium (Thermo Fisher Scientific). Images were captured on an inverted Dmi8 Thunder Imaging System (Leica Microsystems) using the Leica Application Suite X software.

### XGPRT protein structure prediction

Protein structures were predicted locally using AlphaFold3 [116]. Input JSON files were generated for eight sequences across three ligand/enzyme categories: (1) Phosphoribosyltransferase substrates corresponding to PRTase enzymes (InterPro: IPR000836), which catalyze PRPP-dependent salvage reactions using adenine, guanine, hypoxanthine, uracil, orotate, or xanthine together with PRPP and Mg²⁺. This group includes the enzyme classes APRT (adenine phosphoribosyltransferase), HGPRT (hypoxanthine-guanine phosphoribosyltransferase), HPRT (hypoxanthine phosphoribosyltransferase), OPRT (orotate phosphoribosyltransferase), UPRT (uracil phosphoribosyltransferase), XGPRT (xanthine phosphoribosyltransferase), and their shared co-substrate PRPP. (2) TCA-cycle intermediates and related α-oxo/α-hydroxy acids, including citrate (CIT), malate (MLT), oxaloacetate (OAA), α-ketoglutarate (AKG), glyoxylate (GXO), and hydroxypyruvate (HPV), all of which bind Mg²⁺ in relevant metabolic reactions. Enzyme classes associated with these ligands are CS (citrate synthase), ME (malic enzyme), and SCS (succinyl-CoA synthetase). (3) Nucleotide- and phosphate-transfer reaction substrates, comprising ATP, ADP, AMP, GDP, GTP, phosphoenolpyruvate (PEP), 3-phosphoglycerate (3-PG), succinate, CoA, and NAD(P), each accompanied by Mg²⁺ where required. Enzyme classes include AK (adenylate kinase), NDK (nucleoside diphosphate kinase), PEPCK (phosphoenolpyruvate carboxykinase), PGK (phosphoglycerate kinase), PK (pyruvate kinase), and PurF (amidophosphoribosyltransferase). For cases where the oligomeric state of a complex could not be reliably inferred from experimental structures, both dimeric and tetrameric assemblies were modeled.

### XGPRT protein structure analysis

Prediction confidence was assessed using multiple metrics extracted from AlphaFold3 outputs: interface predicted template modeling score (ipTM), overall and binding pocket per-residue confidence scores (predicted local difference distance test = pLDDT), predicted aligned error (PAE) of the ligands, and protein-ligand contact probability. For binding pocket analysis, including ligand-binding pocket PAE, residues were defined as those with ≥3 heavy atoms within 4 Å of substrate ligands. All-atom contacts within 5 Å of ligands were identified using custom Python scripts. Data analysis was performed using Python 3.13.1 with NumPy 2.2.2, Pandas 2.2.3, Matplotlib 3.10.0, and Biopython 1.85. Structures were visualized using PyMOL v3.10 [117]. All visualizations used consistent orientations achieved by structural alignment of protein backbones (Cα atoms) with root mean square deviation (RMSD) 0.040–0.133 Å. Structural conservation was visualized using SSDraw [118].

## Availability of source code and requirements

Project name: HGT in ants

Project homepages: https://github.com/janina-rinke/HGT_in_ants.git -main analysis code https://github.com/dinhe878/GAGA-Metagenome-LGT -automatic HGT detection pipeline

https://github.com/ArsLeicholt/HGT_structural_analysis -protein structure analysis

License: GPL-3.0 (main analysis code and automatic HGT detection pipeline), MIT (protein structure analysis)

Operating system: Linux, macOS, Windows

Programming language: Bash, Python, R, Perl

Hardware requirements: None

## Additional files

**Figure S1:** Synteny analysis of recurrent HGT candidates across ant genomes outside ofthe clade-specific HGTs. Related to Figure 1.

**Figure S2:** Gene Ontology (GO) enrichment analysis of all 497 inferred HGT candidates. Related to Figure 1.

**Figure S3:** Distribution and diversity of Ankyrin-repeat (ANK) domain-containing HGT loci across the GAGA ant phylogeny. Related to Figure 1.

**Figure S4:** Regression analysis of ANK gene expression levels against sequence identity to bacterial reference proteins. Related to Figure 1.

**Figure S5:** Lysozyme gene models inferred from StringTie. Related to Figure 2A.

**Figure S6:** Gene tree of full-length CFA synthases within the Formicini tribe together with expression information. Related to Figure 1.

**Figure S7:** Synteny visualization of all CFA synthases within the Formicini tribe. Related to Figure 1.

**Figure S8:** RNAseq coverage patterns in the proximity of six HGT loci presented in Figure 3.

**Figure S9:** XGPRT expression in *Cardiocondyla obscurior* adults. Related to Figure 4.

**Figure S10:** KEGG pathway enrichment cnetplot of co-expressed genes. Related to Figure 4.

**Figure S11:** Comparison of AlphaFold3 substrate-binding predictions across bacterial XGPRT homologs. Related to Figure 4.

**Figure S12:** AlphaFold3 prediction metrics for binding of TCA-cycle and -oxo-acid substrates to XGPRT homologs. Related to Figure 4.

**Figure S13:** AlphaFold3 multimetric evaluation of canonical phosphoribosyltransferase (PRTase) substrates binding to XGPRT homologs. Related to Figure 4.

**Figure S14:** Structural conservation of the XGPRT fold across bacterial homologs with the conserved loop region highlighted. Related to Figure 4.

**Figure S15:** Genomic evidence supporting a putative horizontal gene transfer (HGT) event of a Xanthine-guanine-phosphoribosyltransferase (XGPRT) on Scaffold8 in GAGA-0515 (*Cardiocondyla obscurior*). Related to Material & Methods.

**Figure S16:** Genomic evidence supporting a putative horizontal gene transfer (HGT) event of a lysozyme gene on Scaffold20 in GAGA-0099 (*Leptothorax acervorum*). Related to Material & Methods.

**Figure S17:** Genomic evidence supporting a putative horizontal gene transfer (HGT) event of a Cyclopropane fatty acidy synthase gene on Scaffold6 in GAGA-0485 (*Liometopum microcephalum*). Related to Material & Methods.

**Figure S18:** Genomic evidence supporting a putative horizontal gene transfer (HGT) event of an ANK repeat gene on Scaffold15 in GAGA-0087 (*Myrmica scabrinoides*). Related to Material & Methods.

**Figure S19:** Parameters used for automated filtering of HGT candidates predicted by the HGT finder pipeline. Related to Material & Methods.

**Figure S20:** Testing for XGPRT correlation to *Westeberhardia* gene expression. Related to Material & Methods.

**Figure S21:** Representative HCR-FISH Image demonstrating low and diffuse background signal for the Alexa Fluor 647 in the absence of expression specificity. Related to Material & Methods.

Table_S1_HGTs_all_info.xlsx.

Table_S2_PCR_validations.xlsx.

Table_S3_Comparison_previous_HGTs_ants.xlsx.

Table_S4_Synteny_other_HGT_loci.xlsx.

Table_S5_Lysozyme_HGTs.xlsx.

Table_S6_MurNAc_HGTs.xlsx.

Table_S7_CFA_HGTs.xlsx.

Table_S8_In-depth_Other_HGTs_summary.xlsx.

Table_S9_kegg_enrichment.tsv.

Table_S10_GAGA_species_list.txt.

Table_S11_PATRIC_genome_list_21112020.xlsx.

Table_S12_Insect_Assembly_Acc.xlsx.

Table_S13_GAGA.HGTs.afterFil.preCuration.xlsx.

Table_S14_Correlation_Results_HGT RNAseq.xlsx.

## Supplementary Material

giag043_Supplemental_Files

giag043_Authors_Response_To_Reviewer_Comments_Original_Submission

giag043_GIGA-D-26-00041_Original_Submission

giag043_GIGA-D-26-00041_Revision_1

giag043_Reviewer_1_Report_Original_Submission Reviewer 1 -- 3/1/2026

giag043_Reviewer_2_Report_Original_Submission Reviewer 2 -- 3/3/2026

## Data Availability

The datasets supporting the conclusions of this article are included in the article and its additional files. All files and HGT candidate sequences, as well as gene annotation files are available in the Figshare repository [119].
