# Supplementary material for: Comparative analysis of 163 ant genomes reveals recurrent horizontal gene transfer from bacteria to ants
Source: Gigascience. 2026 Apr 6;15:giag043. doi: 10.1093/gigascience/giag043 (PMC13320244; doi:10.1093/gigascience/giag043)
Supplement: giag043_Supplemental_Files [file giag043_supplemental_files.zip › HGTinAnts_Supplementary.pdf]

Online Supplement: Horizontal Gene Transfer in Ants

Janina Rinke[1] Lukas Franke[1] Ding He[2] Maike Fischer[3] Joel Vizueta[2]
Lars A. Eicholt[1] Rasmus Stenbak Larsen[2] Zijun Xiong[4]
Phoebe H. M. Cunningham[5] Lee Henry[5] Martin Kaltenpoth[3] Jürgen Gadau[1]
Guojie Zhang[2,6] Jacobus J. Boomsma[2] Lukas Schrader[1*]

[1]University of Münster, Münster, Germany
[2]University of Copenhagen, Copenhagen, Denmark
[3]Max-Planck-Institut für chemische Ökologie, DE-07745 Jena, Germany
[4]Nanchang University, Nanchang, China
[5]Queen Mary University London, London E1 4NS, United Kingdom
[6]Zhejiang University, Hangzhou, China

*Corresponding author; E-mail: lukas.schrader@uni-muenster.de

Supplementary Figures

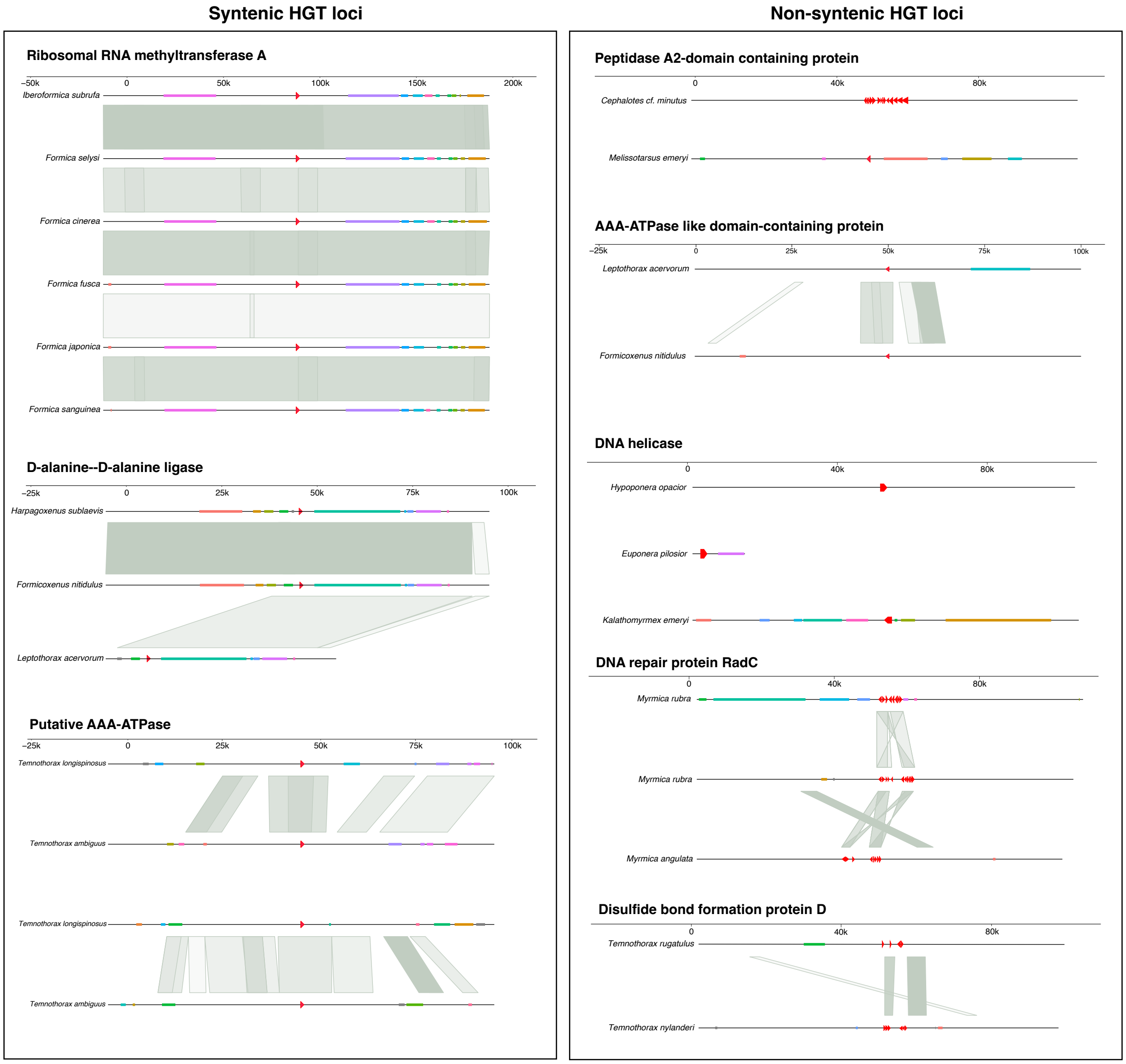

Figure S1. Synteny analysis of recurrent HGT candidates across ant genomes outside of the clade-specific HGTs. Related to Figure 1. HGT loci were extended by 50 kb upstream and downstream to assess local genomic context and conservation of synteny among ant species. *(Left panel)* Three HGT candidates exhibit conserved synteny, with homologous flanking regions indicating stable genomic integration and conserved neighborhood structure across taxa. *(Right panel)* Five HGT candidates lack conserved synteny, with flanking regions showing low or no homology across species, suggesting either more recent insertions, lineage-specific rearrangements, or independent acquisition events. Colored blocks represent protein-coding genes; orthologous genes across species are shown in matching colors, while focal HGTs are highlighted in red. Scaffold coordinates and gene annotations are provided in Supplemental Table S4.

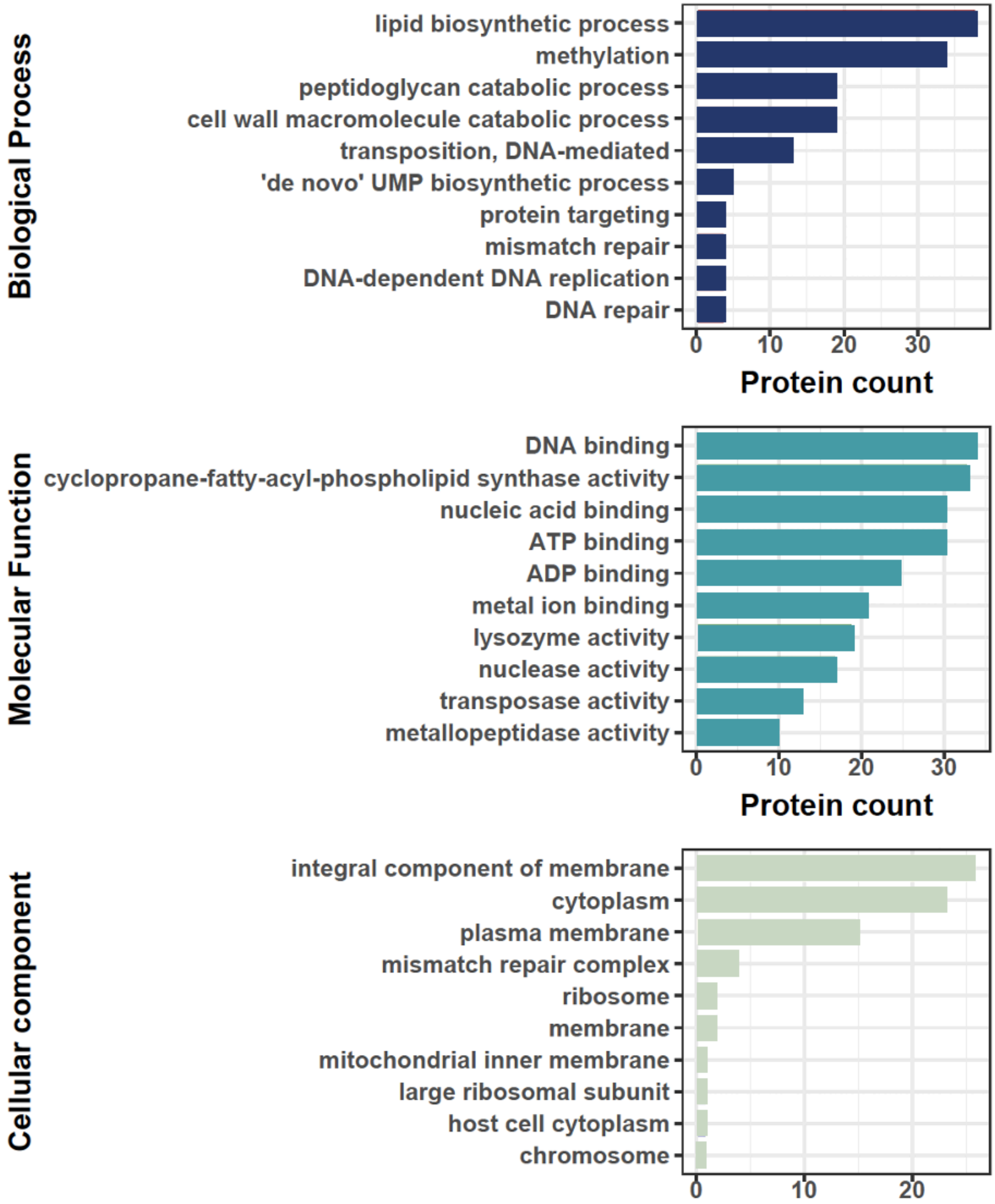

Figure S2. Gene Ontology (GO) enrichment analysis of all 497 inferred HGT candidates. Related to Figure 1. GO terms associated with all predicted HGT candidate proteins were inferred using the *UniProtR* package in R. Enrichment was assessed across three GO categories: Biological Process (BP), Molecular Function (MF), and Cellular Component (CC). Bars represent the number of HGT candidate proteins associated with each significantly enriched GO term. This analysis provides insight into the potential functional roles of HGT-derived proteins within recipient ant genomes. Full GO annotations and associated protein IDs are provided in Supplemental Table S1.

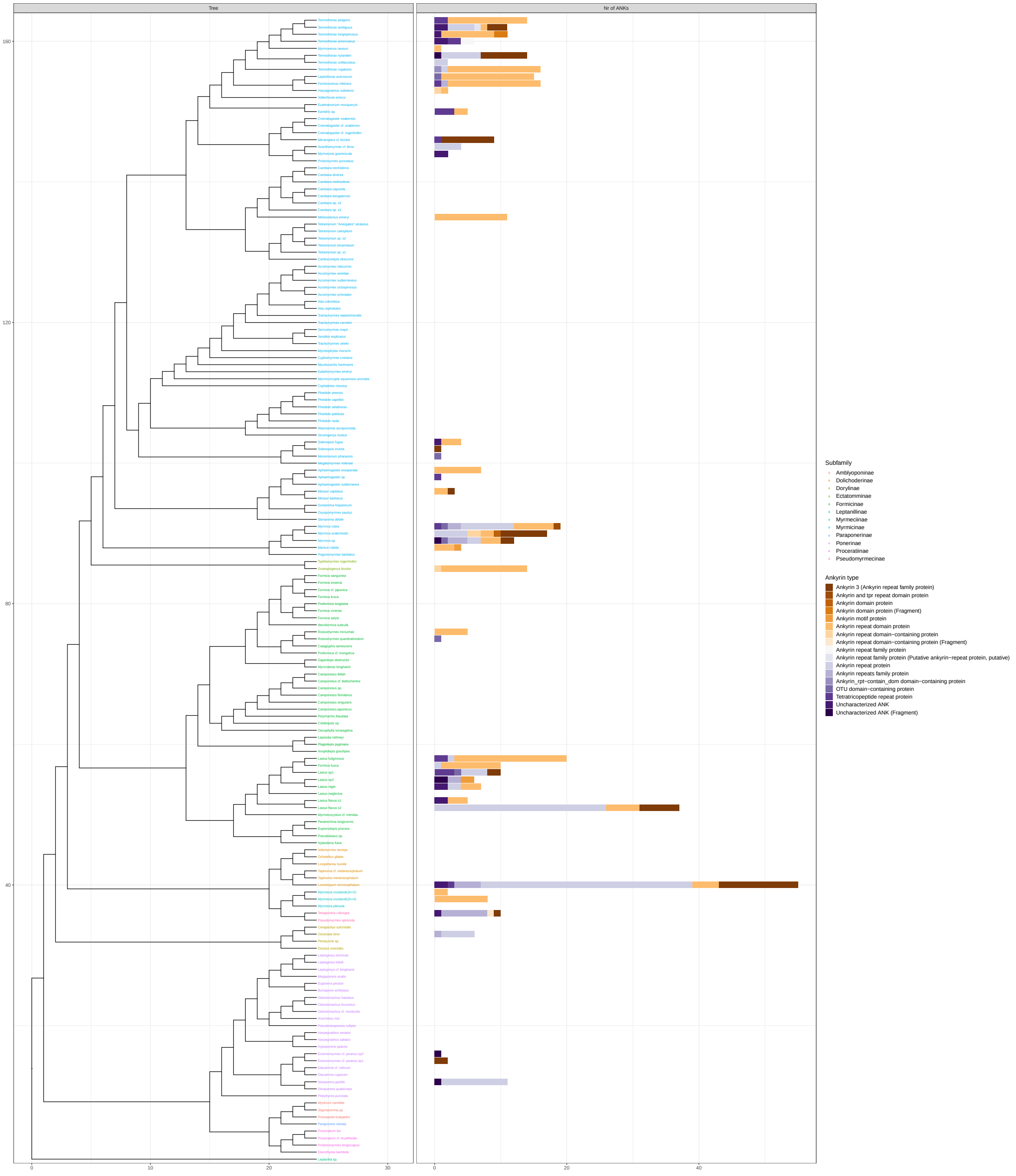

Figure S3. Distribution and diversity of Ankyrin-repeat (ANK) domain-containing HGT loci across the GAGA ant phylogeny. Related to Figure 1. The number and types of ANK HGT loci are shown for each of the 163 ant species included in this study. Species are arranged according to the ant phylogeny and colored by their respective subfamily. ANK genes are categorized into distinct Ankyrin types based on UniRef cluster assignments and prokaryotic gene annotation data. The visualization highlights both the widespread occurrence and lineage-specific patterns of ANK HGTs across ants.

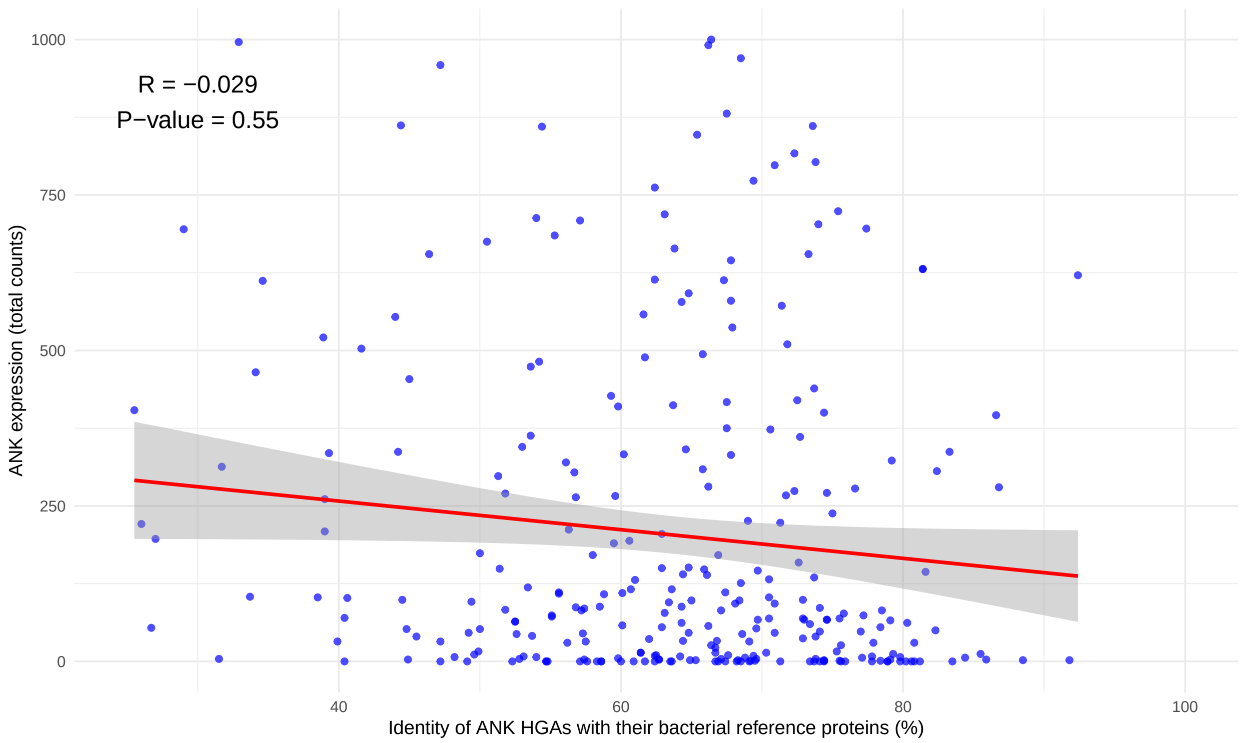

Figure S4. Regression analysis of ANK gene expression levels against sequence identity to bacterial reference proteins. Related to Figure 1. Each data point represents an ANK HGT candidate, plotting its expression (RNA-seq read counts) against its percentage identity to the closest bacterial reference protein. No significant correlation was observed (R = –0.029, p = 0.55), suggesting that ANK gene expression in ant genomes is not predicted by sequence similarity to the original bacterial donors.

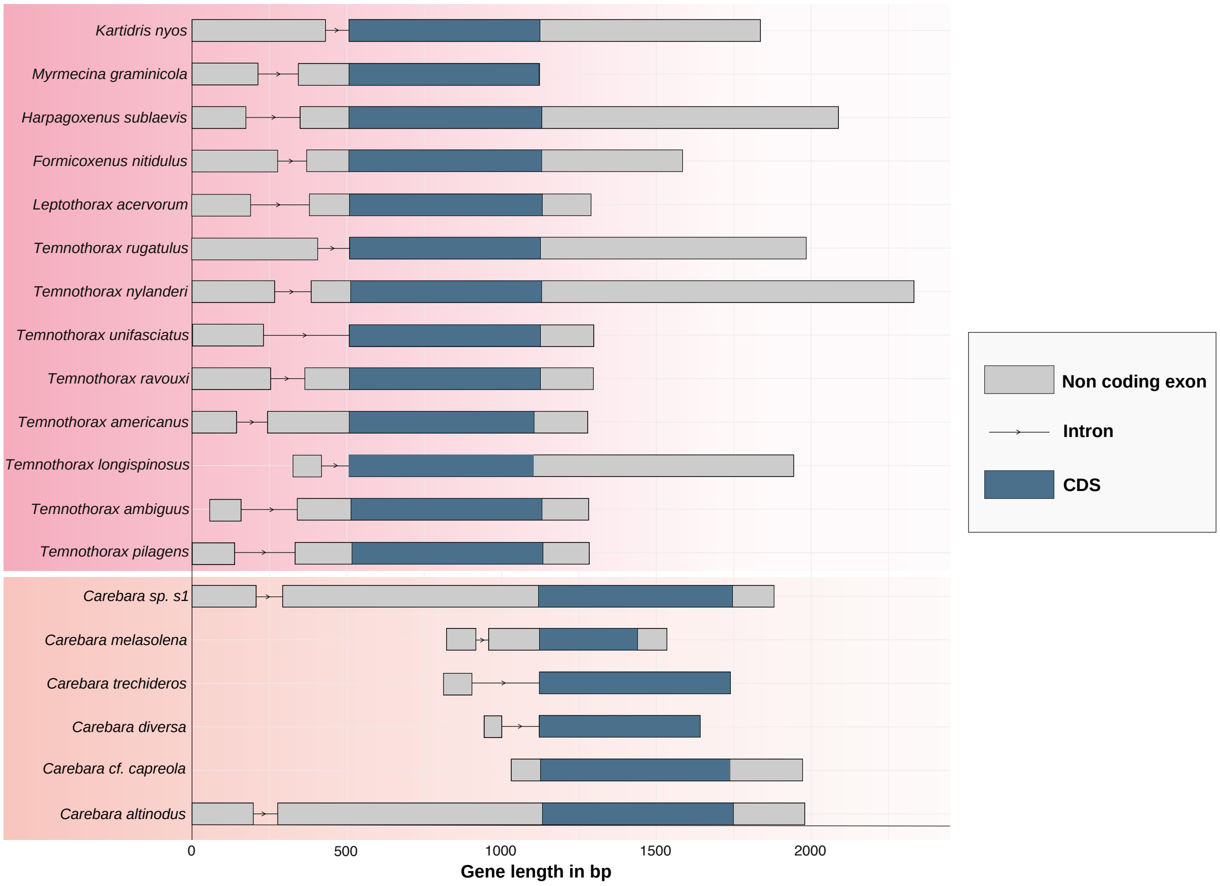

Figure S5. Lysozyme gene models inferred from StringTie. Related to Figure 2A. Predicted gene structures for lysozyme HGT loci are shown for representative species of the *Carebara* and *Temnothorax et al.* clades. Gene models were generated using StringTie and are visualized with non-coding exons in grey, coding sequences (CDS) in blue, and introns as black lines with arrows indicating transcriptional direction. Gene architectures differ notably between clades, reflecting divergent splicing patterns or gene structures associated with each evolutionary lineage.

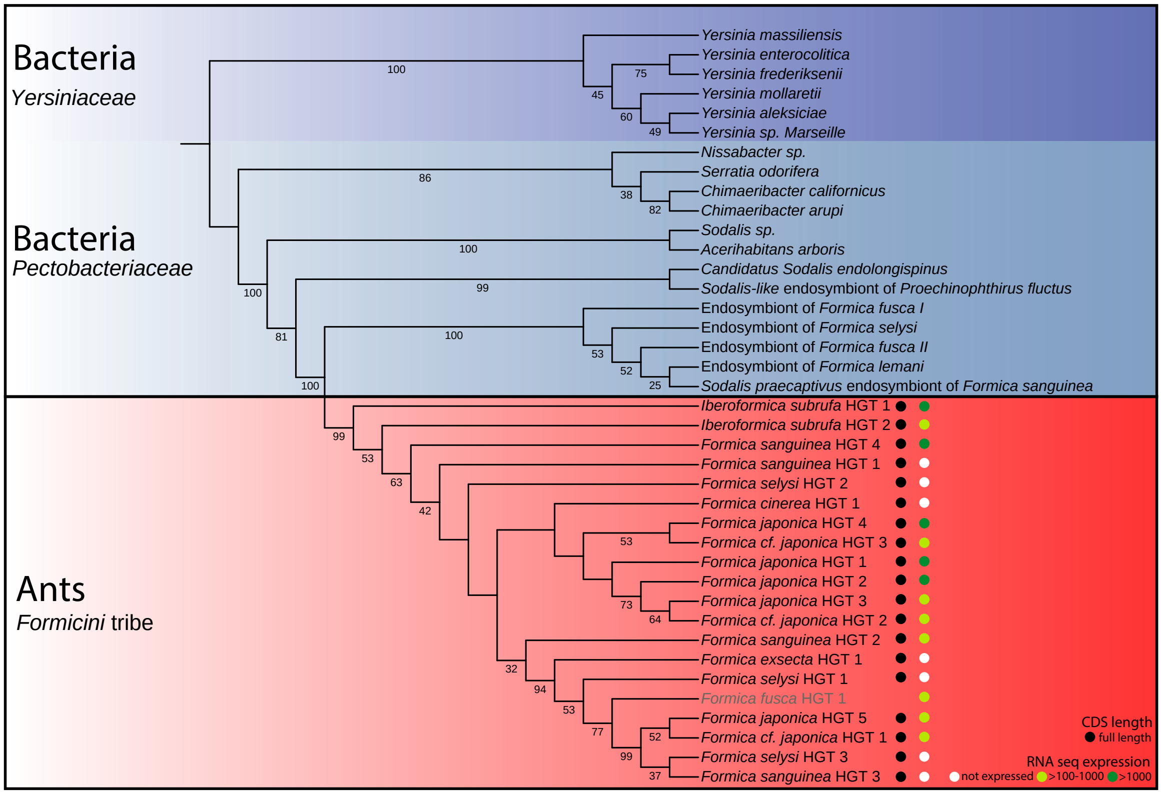

Figure S6. Gene tree of full-length CFA synthases within the Formicini tribe together with expression information. Related to Figure 1. Phylogenetic reconstruction of all detected Cyclopropane Fatty Acid (CFA) synthase gene loci identified in Formicini ants. The tree was inferred from protein sequences and includes the best BLAST hits for each CFA gene. Black dots indicate full-length CFA synthase coding sequences (CDS), while green dots denote expression support based on RNA-seq data.

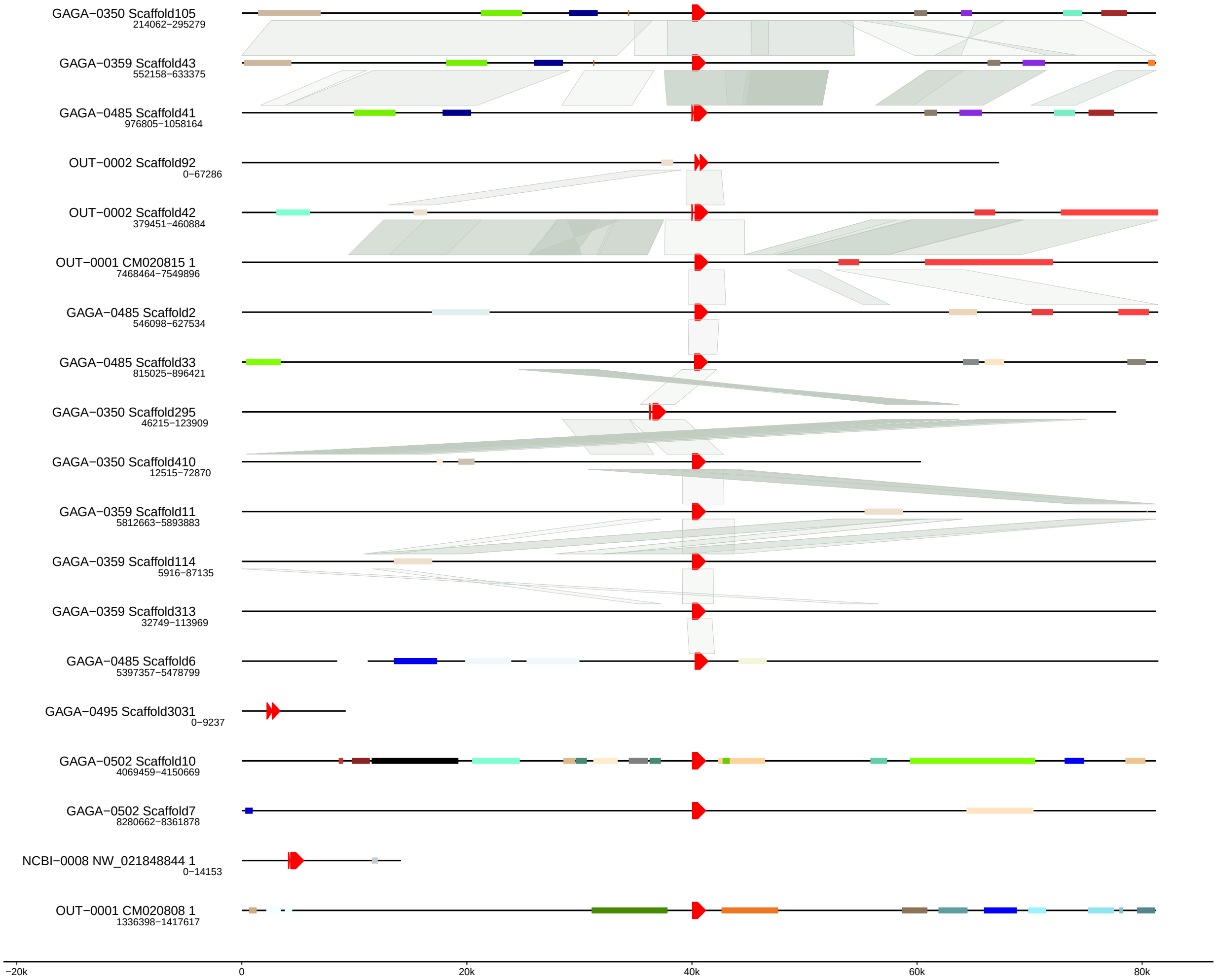

Figure S7. Synteny visualization of all CFA synthases within the Formicini tribe. Related to Figure 1. Genomic context of putative CFA synthase HGT loci across Formicini ants, visualized with 50 kb extensions upstream and downstream of each HGT locus. Colored bars represent annotated genes, with identical colors indicating putative orthologs based on gene identity and position. The focal CFA synthase HGT is shown in red. Synteny is not consistently conserved across species, highlighting variability in the genomic neighborhoods surrounding CFA loci.

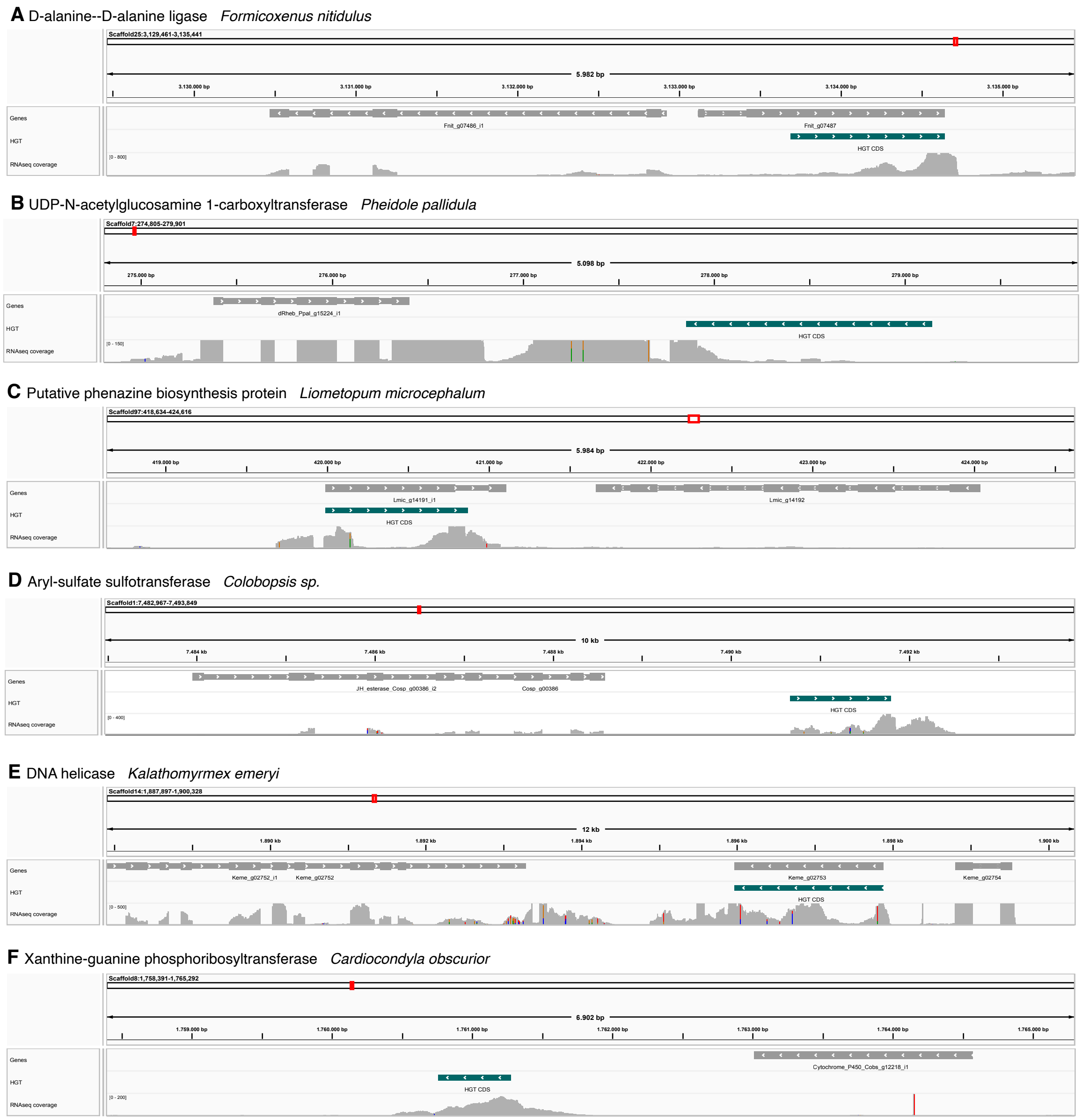

Figure S8. RNAseq coverage patterns in the proximity of six HGT loci presented in Figure 3. Each panel shows, from top to bottom, annotated gene models from Vizueta et al. 2025, our annotated HGT CDS, and RNAseq coverage tracks for (A) a D-alanine–D-alanine ligase HGT in *Formicoxenus nitidulus* (B) an UDP-N-acetylglucosamine 1-carboxyltransferase HGT in *Pheidole pallidula* (C) a putative phenazine biosynthesis protein in *Liometopum microcephalum* (D) an Aryl-sulfate sulfotransferase in *Colobopsis sp.* (E) a DNA helicase in *Kalathomyrmex emeryi* and (F) a Xanthine Guanine Phosphoribosyltransferase in *Cardiocondyla obscurior*. Coverage tracks were produced by mapping samples of available short-read RNAseq data from each species (see Vizueta et al. 2025) against the respective reference genome with STAR (A,C,E,F) or HiSat2 (B,D). Note that the annotations from Vizueta et al. 2025 overlapping with HGT loci (Fnit_g07487 in A, Keme_g02753 in E) are consistent with the inferred bacterial origin of these genes. The closest homolog to Fnit_g07487 is "MAG: D-alanine–D-alanine ligase A" from a *Sodalis* bacterium (Genbank Accession: UVK77028.1). The closest homolog to Keme_g02753 is "ATP-dependent helicase" from a *Wolbachia* bacterium (Genbank Accession: WP_174133902.1).

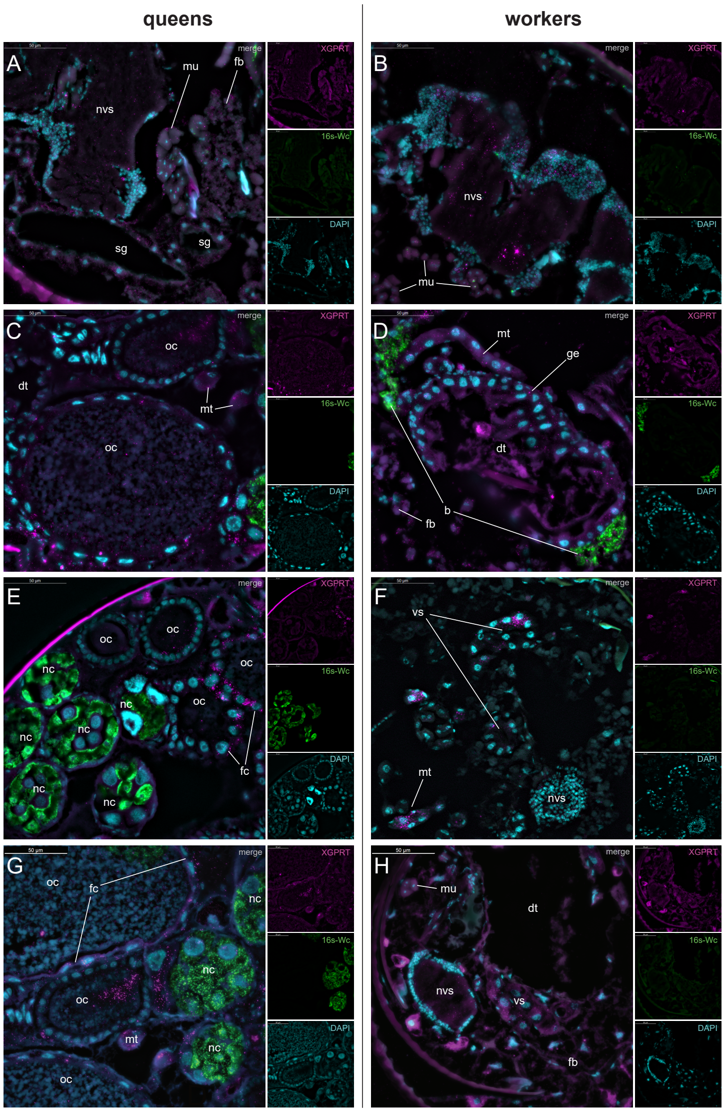

Figure S9: XGPRT expression in *Cardiocondyla obscurior* adults. Related to Figure 4. mRNA of XGPRT (magenta) and 16S rRNA of *Westeberhardia cardiocondylae* (16S-Wc, green) were fluorescently labelled using HCR-FISH in sections of *C. obscurior* queens (A, C, E, G) and workers (B, D, F, H). Host nuclei were counterstained with DAPI (cyan). Each panel shows the main overlay image with the three corresponding single-channel images. b = bacteriome; fb = fat body; fc= follicle cell; dt = digestive tract; ge = gut epithelium; mu = muscle; mt = malpighian tubule; nc = nurse cell, nvs = nervous system; oc = oocyte; sg = salivary gland; vs = venom system.

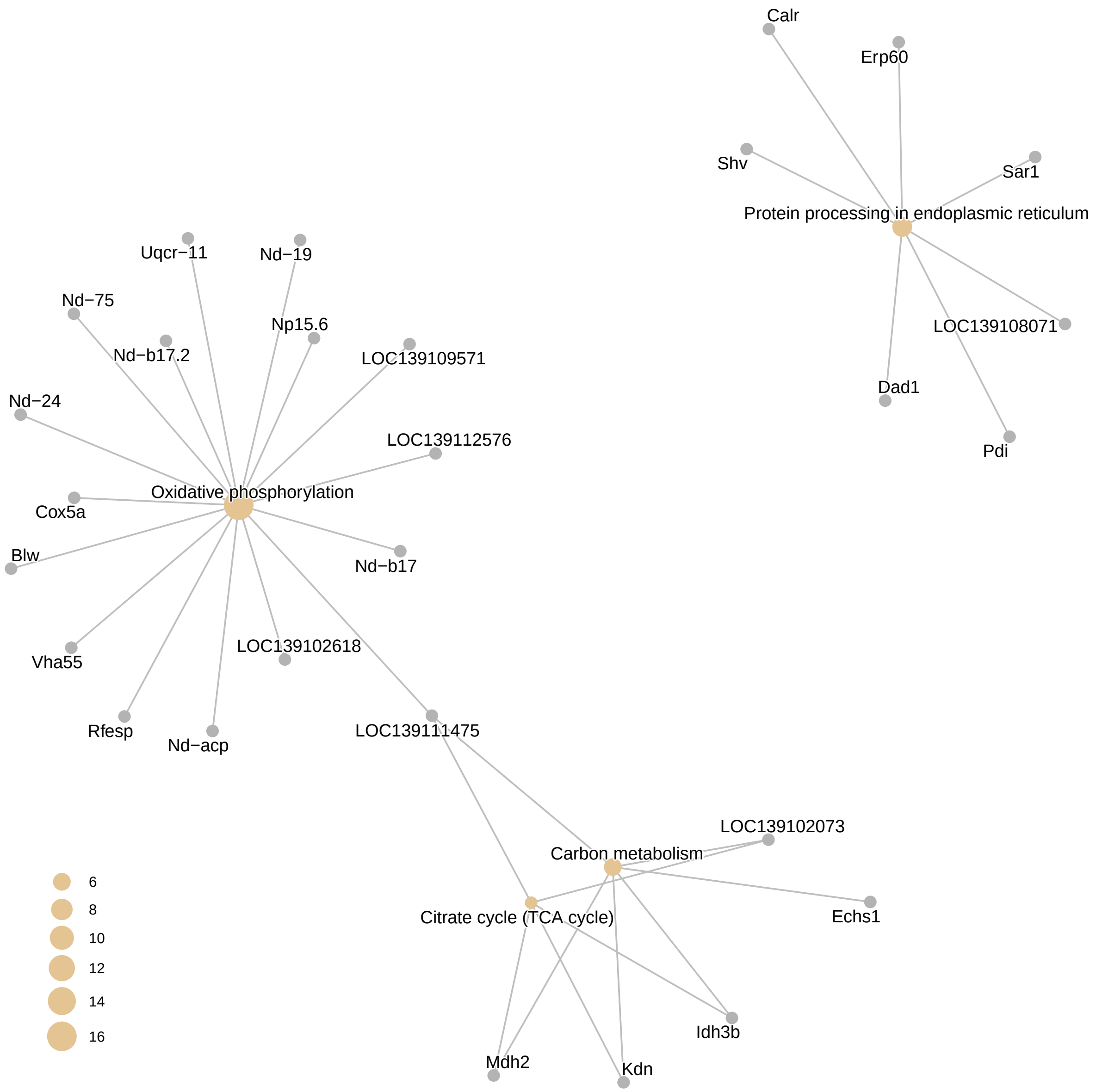

Figure S10: KEGG pathway enrichment cnetplot of co-expressed genes. Related to Figure 4. The cnetplot illustrates KEGG pathways significantly enriched among genes co-expressed with the horizontally transferred XGPRT in *Cardiocondyla obscurior*. Nodes represent individual genes (circles) and enriched KEGG pathways (larger labeled nodes), with edges indicating gene–pathway associations. Pathway node size corresponds to the number of genes contributing to each KEGG term.

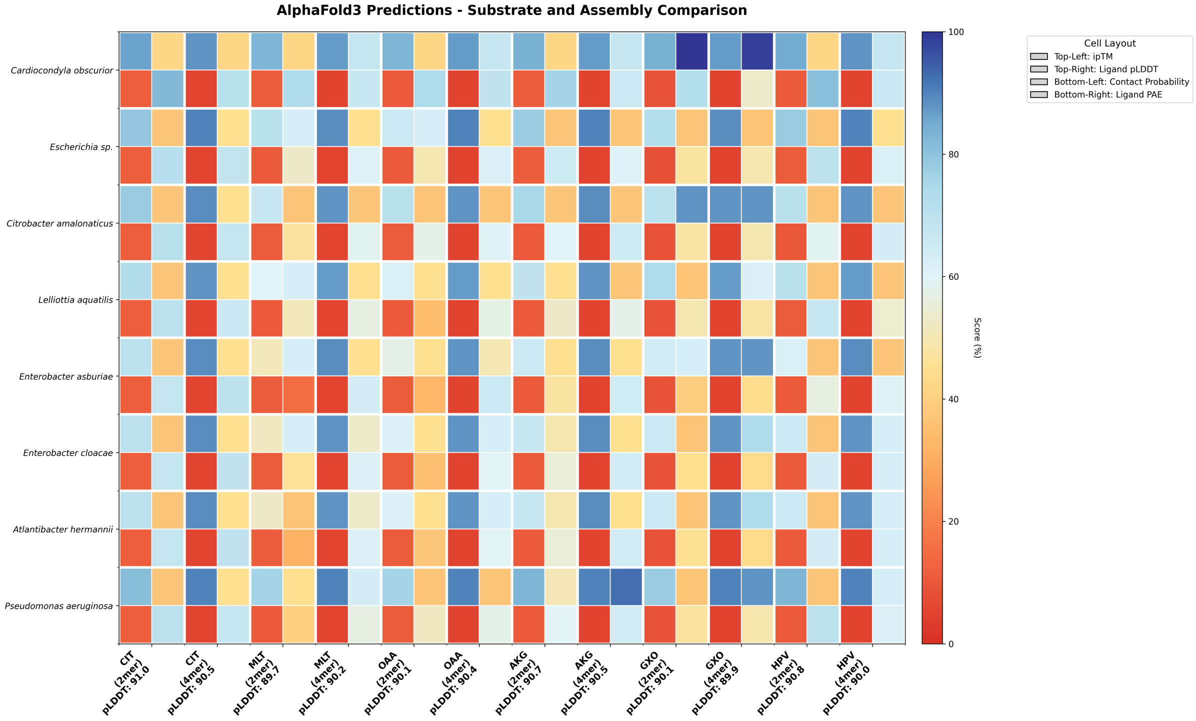

Figure S11: Comparison of AlphaFold3 substrate-binding predictions across bacterial XGPRT homologs. Related to Figure 4. Heatmap summarizing four AlphaFold3 confidence metrics for each protein–substrate pair. Each tile is divided into four quadrants: top-left = ipTM, top-right = ligand pLDDT, bottom-left = protein–ligand contact probability, and bottom-right = ligand–to–protein PAE (scaled to 0–100%). Rows correspond to XGPRT homologs from different bacterial taxa, and columns represent distinct substrates. Higher (bluer) scores reflect more confident binding metrices. Substrate names are annotated with the predicted oligomeric assembly (“2mer” or “4mer”) and mean protein pLDDT.

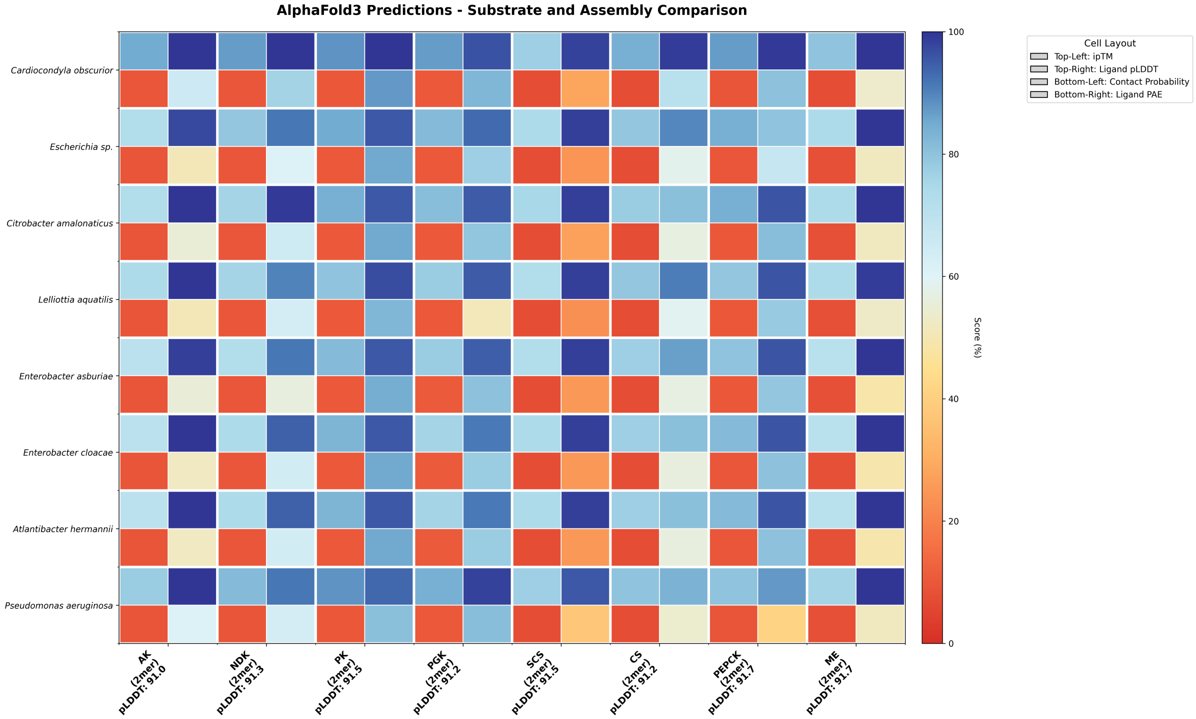

Figure S12: AlphaFold3 prediction metrics for binding of TCA-cycle and -oxo-acid substrates to XGPRT homologs. Related to Figure 4. Heatmap showing ipTM, ligand pLDDT, contact probability, and ligand PAE for each homolog–substrate combination. Each square is partitioned into four quadrants (top-left: ipTM; top-right: ligand pLDDT; bottom-left: contact probability; bottom-right: ligand PAE), scaled to a 0–100% color range. Rows represent homologs from diverse Enterobacteriaceae and related taxa; columns show predictions for different TCA-cycle intermediates and -oxo acids. Labels indicate multimeric assembly used during prediction and the mean pLDDT of the protein model. Higher (bluer) scores reflect more confident binding metrices.

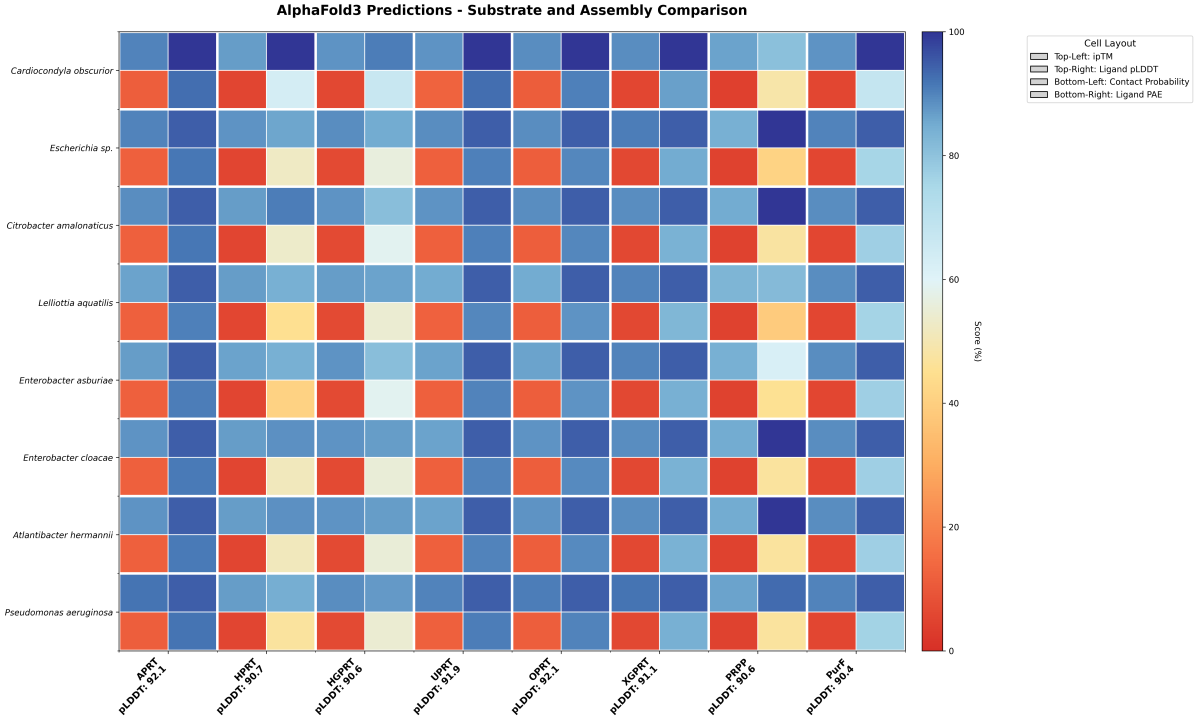

Figure S13: AlphaFold3 multimetric evaluation of canonical phosphoribosyltransferase (PRTase) substrates binding to XGPRT homologs. Related to Figure 4. Heatmap displaying four AlphaFold3 confidence measures—ipTM, ligand pLDDT, protein–ligand contact probability, and ligand PAE—summarized in quadrant layout within each tile. Homologs are arranged by taxonomy (rows), and substrates include APRT, HPRT, XGPRT, OPRT, UPRT, PRPP, and PurF (columns). Substrate labels include mean protein pLDDT. Higher (bluer) scores reflect more confident binding metrices.

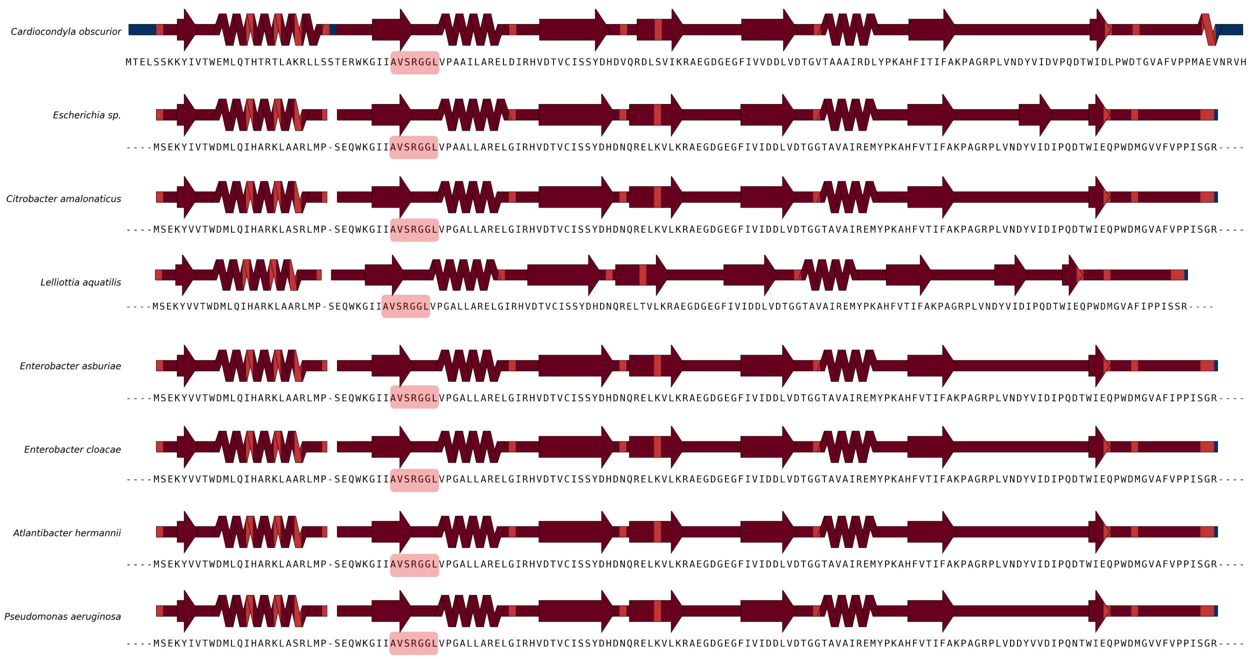

Figure S14: Structural conservation of the XGPRT fold across bacterial homologs with the conserved loop region highlighted. Related to Figure 4. Secondary-structure schematics generated with SSdraw show the conserved domain architecture of XGPRT homologs from *Cardiocondyla obscurior* and representative -Proteobacteria. Helices are shown as coils and -strands as arrows, aligned to the underlying sequence. The functionally relevant loop region is highlighted in red, illustrating its strong positional and structural conservation across all homologs despite minor sequence variation. This loop corresponds to the binding-site–proximal segment implicated in ligand coordination in the *C. obscurior* enzyme.

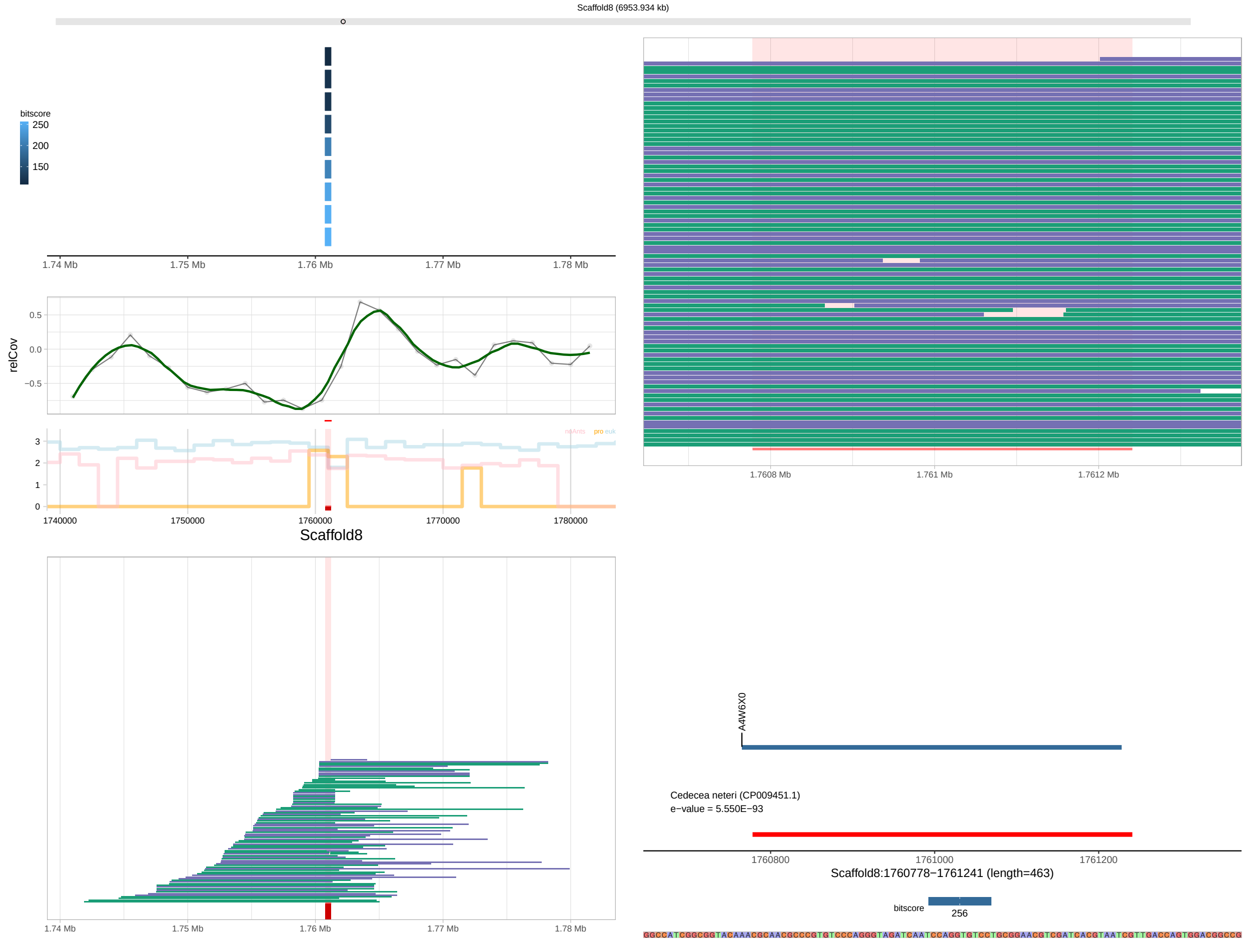

Figure S15. Genomic evidence supporting a putative horizontal gene transfer (HGT) event of a Xanthine-guanine-phosphoribosyltransferase (XGPRT) on Scaffold8 in GAGA-0515 (*Cardiocondyla obscurior*). Related to Material & Methods. *(Top)* Schematic representation of Scaffold8, with the HGT candidate region highlighted in red (not visible at this scale; see zoomed-in view in PDF).

(Left panels) • **1st panel:** Close-up of the candidate region (red highlight) and flanking sequences. Blue boxes indicate significant matches (E-value < 1e-5) to prokaryotic proteins in the reference database (n = 9 hits, colored according to their bitscore). • **2nd panel:** Log2-scaled relative coverage from PacBio long-read genomic data. Values near 0 indicate coverage consistent with the genomic average; strong deviations (|log2| > 1) may suggest assembly artifacts. • **3rd panel:** log10-scaled bitscores against prokaryotic (orange) and eukaryotic (with ants in blue, without ants in red) databases. The HGT candidate (red box, positions 1760778–1761241) shows a prokaryotic homology spike amid eukaryotic background signals. • **4th panel:** PacBio read alignments spanning the candidate region. True HGTs are supported by reads bridging ancestral DNA (antDNA) and the putative HGT, whereas misassemblies exhibit poor read overlap at boundaries.

(Right panels) • **1st panel:** Detailed view of PacBio reads aligned to the HGT candidate. • **2nd panel:** Best BLAST hit against SwissProt proteins (A4W6X0, blue) and a high-confidence bacterial hit against the bacterial database (*Cedecea neteri*, CP009451.1; E-value = 5.50e–93, red). • **3rd panel:** Nucleotide sequence of the first 75 bp of the HGT candidate.

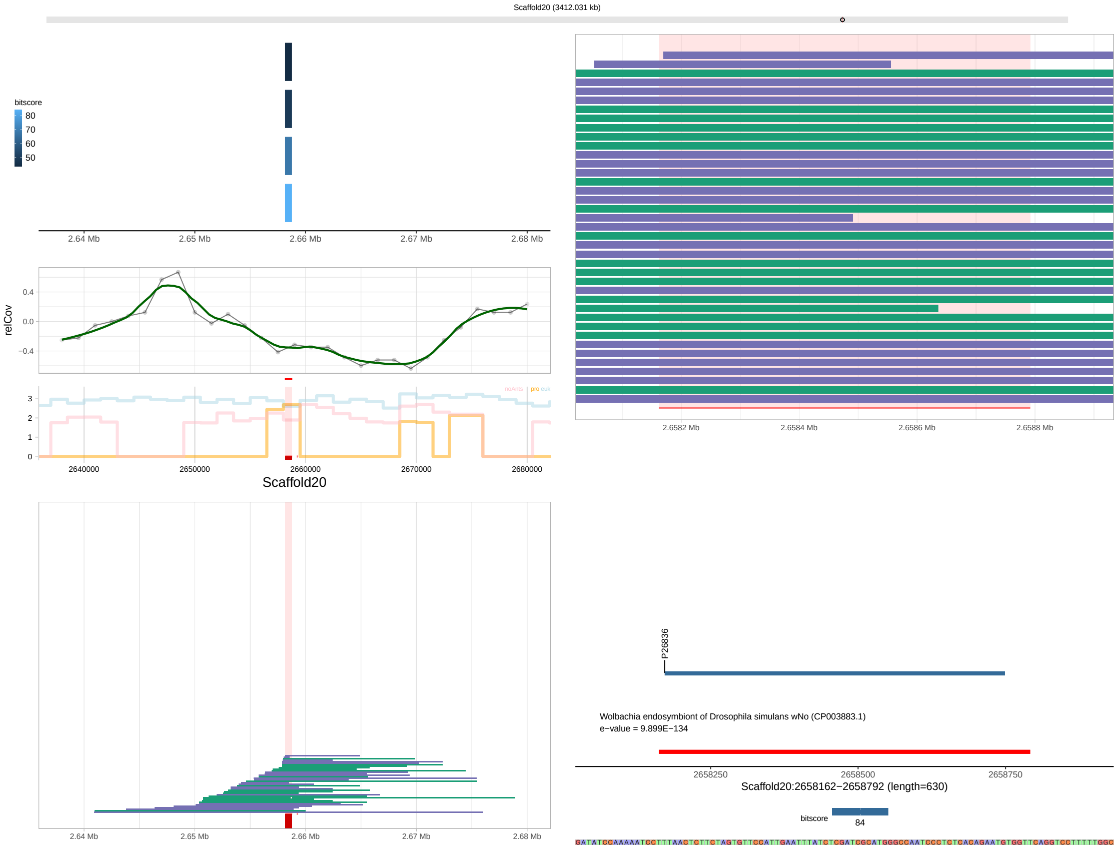

Figure S16. Genomic evidence supporting a putative horizontal gene transfer (HGT) event of a lysozyme gene on Scaffold20 in GAGA-0099 (*Leptothorax acervorum*). Related to Material & Methods. *(Top)* Schematic representation of Scaffold8, with the Lysozyme HGT candidate region highlighted in red (not visible at this scale; see zoomed-in view in PDF).

(Left panels) • **1st panel:** Close-up of the candidate region (red highlight) and flanking sequences. Blue boxes indicate significant matches (E-value $<$ 1e-5) to prokaryotic proteins in the reference database (n = 4 hits, colored according to their bitscore). • **2nd panel:** Log2-scaled relative coverage from PacBio long-read genomic data. Values near 0 indicate coverage consistent with the genomic average; strong deviations ($|\log2| > 1$) may suggest assembly artifacts. • **3rd panel:** log10-scaled bitscores against prokaryotic (orange) and eukaryotic (with ants in blue, without ants in red) databases. The HGT candidate (red box, positions 2658162−2658792) shows a prokaryotic homology spike amid eukaryotic background signals. • **4th panel:** PacBio read alignments spanning the candidate region. True HGTs are supported by reads bridging ancestral DNA (antDNA) and the putative HGT, whereas misassemblies exhibit poor read overlap at boundaries.

(Right panels) • **1st panel:** Detailed view of PacBio reads aligned to the HGT candidate. • **2nd panel:** Best BLAST hit against SwissProt proteins (P26836, blue) and a high-confidence bacterial hit against the bacterial database (*Wolbachia endosymbiont of Drosophila simulans wNo*, CP003883.1; E-value = 9.899e–134, red). • **3rd panel:** Nucleotide sequence of the first 75 bp of the HGT candidate.

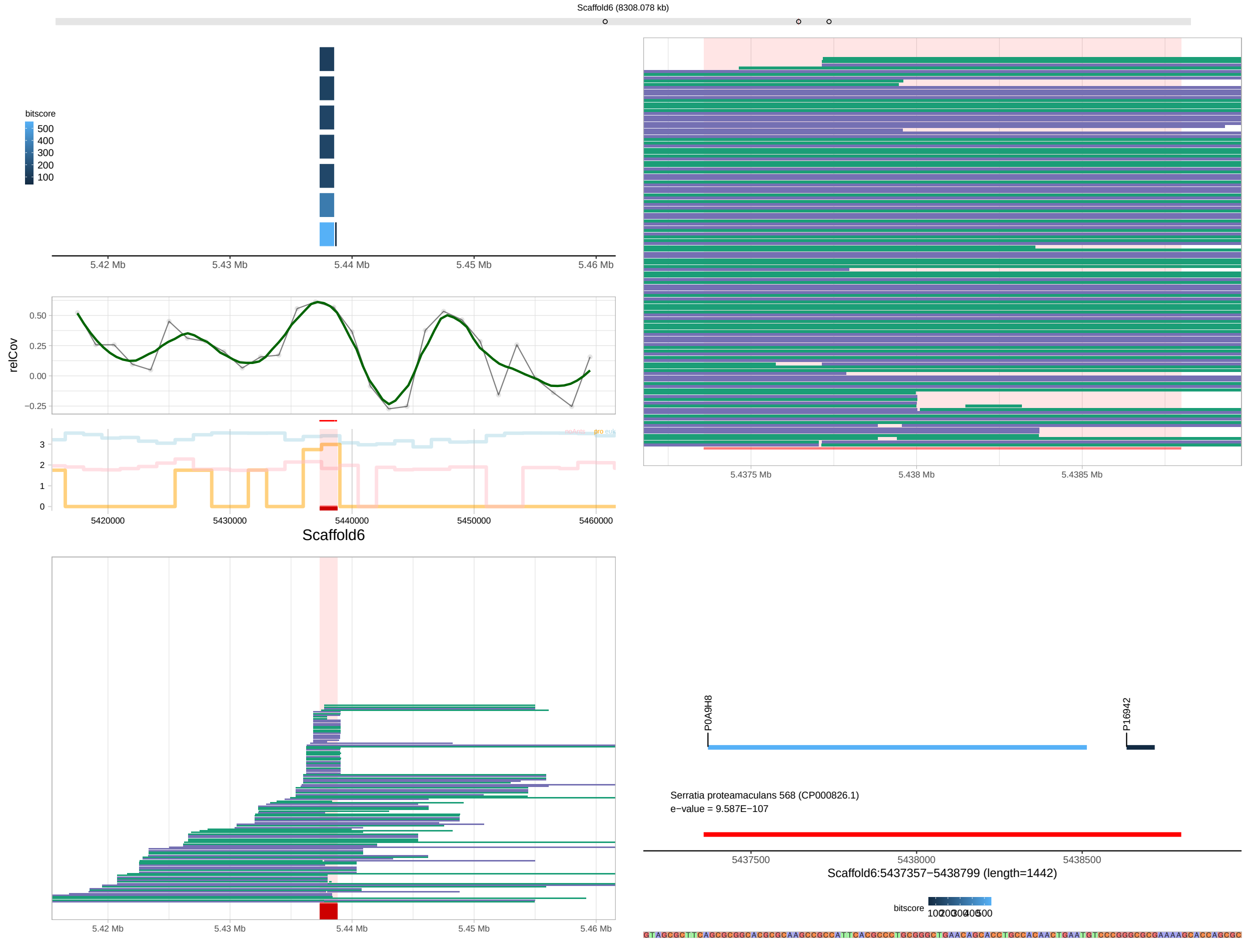

Figure S17. Genomic evidence supporting a putative horizontal gene transfer (HGT) event of a Cyclopropane fatty acidy synthase gene on Scaffold6 in GAGA-0485 (*Liometopum microcephalum*). Related to Material & Methods. *(Top)* Schematic representation of Scaffold6, with the CFA synthase HGT candidate region highlighted in red (not visible at this scale; see zoomed-in view in PDF).

(Left panels) • **1st panel:** Close-up of the candidate region (red highlight) and flanking sequences. Blue boxes indicate significant matches (E-value $<$ 1e-5) to prokaryotic proteins in the reference database (n = 7 hits, colored according to their bitscore). • **2nd panel:** Log2-scaled relative coverage from PacBio long-read genomic data. Values near 0 indicate coverage consistent with the genomic average; strong deviations ($|\log2| > 1$) may suggest assembly artifacts. • **3rd panel:** log10-scaled bitscores against prokaryotic (orange) and eukaryotic (with ants in blue, without ants in red) databases. The HGT candidate (red box, positions 5437357—5438799) shows a prokaryotic homology spike amid eukaryotic background signals. • **4th panel:** PacBio read alignments spanning the candidate region. True HGTs are supported by reads bridging ancestral DNA (antDNA) and the putative HGT, whereas misassemblies exhibit poor read overlap at boundaries.

(Right panels) • **1st panel:** Detailed view of PacBio reads aligned to the HGT candidate. • **2nd panel:** Best BLAST hit against SwissProt proteins (P0A9H8, light blue) and a high-confidence bacterial hit against the bacterial database (*Serratia proteamaculans 568*, CP000826.1; E-value = 9.587e–107, red). • **3rd panel:** Nucleotide sequence of the first 75 bp of the HGT candidate.

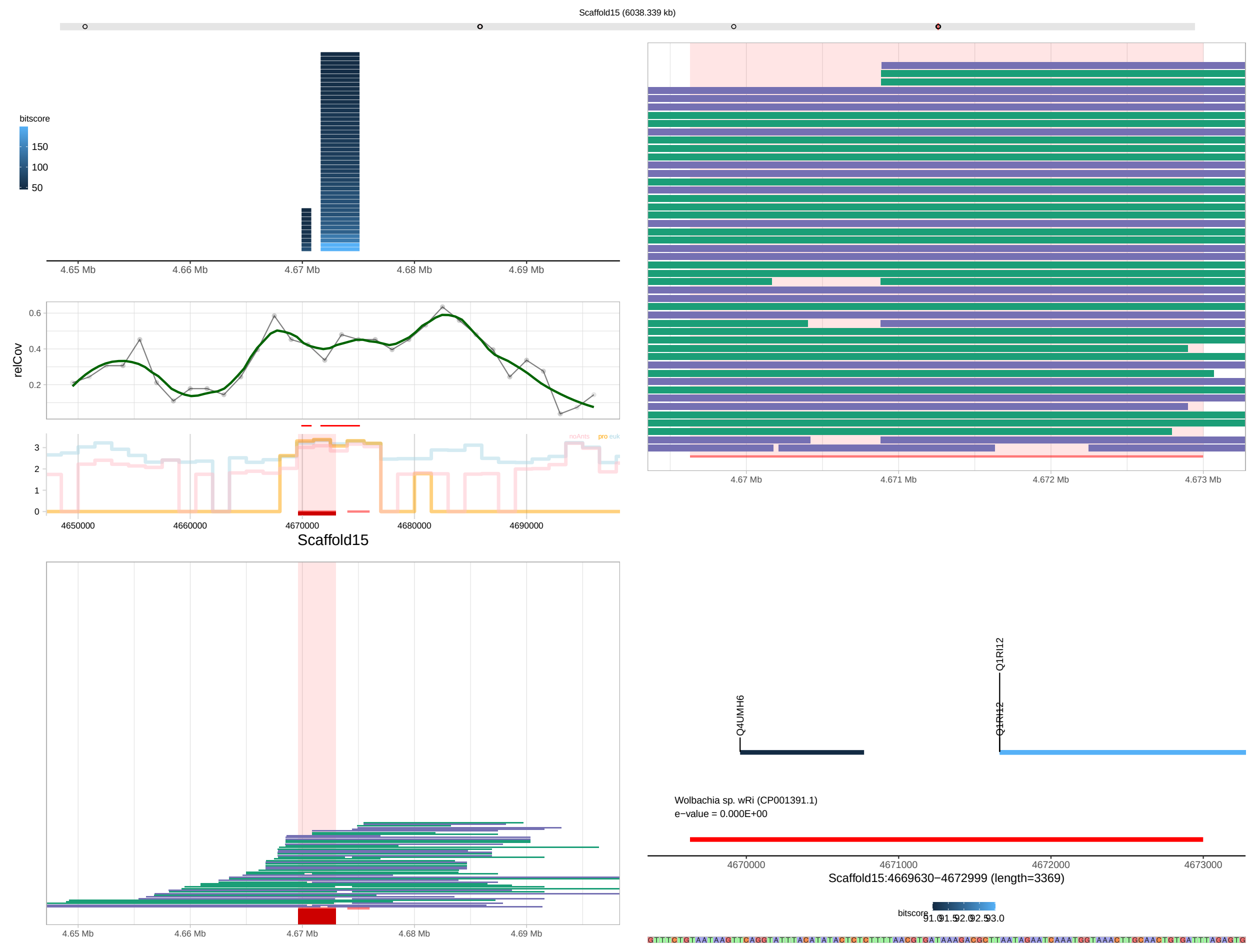

Figure S18. Genomic evidence supporting a putative horizontal gene transfer (HGT) event of an ANK repeat gene on Scaffold15 in GAGA-0087 (*Myrmica scabrinoides*). Related to Material & Methods. *(Top)* Schematic representation of Scaffold15, with the ANK HGT candidate region highlighted in red (not visible at this scale; see zoomed-in view in PDF).

(Left panels) • **1st panel:** Close-up of the candidate region (red highlight) and flanking sequences. Blue boxes indicate significant matches (E-value $<$ 1e-5) to prokaryotic proteins in the reference database (n = 25 hits, colored according to their bitscore). • **2nd panel:** Log2-scaled relative coverage from PacBio long-read genomic data. Values near 0 indicate coverage consistent with the genomic average; strong deviations ($|\log 2| > 1$) may suggest assembly artifacts. • **3rd panel:** log10-scaled bitscores against prokaryotic (orange) and eukaryotic (with ants in blue, without ants in red) databases. The HGT candidate (red box, positions 4669630−4672999) shows a prokaryotic homology spike amid eukaryotic background signals. • **4th panel:** PacBio read alignments spanning the candidate region. True HGTs are supported by reads bridging ancestral DNA (antDNA) and the putative HGT, whereas misassemblies exhibit poor read overlap at boundaries.

(Right panels) • **1st panel:** Detailed view of PacBio reads aligned to the HGT candidate. • **2nd panel:** Best BLAST hit against SwissProt proteins (Q4UMH6, black; Q1RI12, light blue) and a high-confidence bacterial hit against the bacterial database (*Wolbachia sp. wRi*, CP001391.1; E-value = 0.000E+00, red). • **3rd panel:** Nucleotide sequence of the first 75 bp of the HGT candidate.

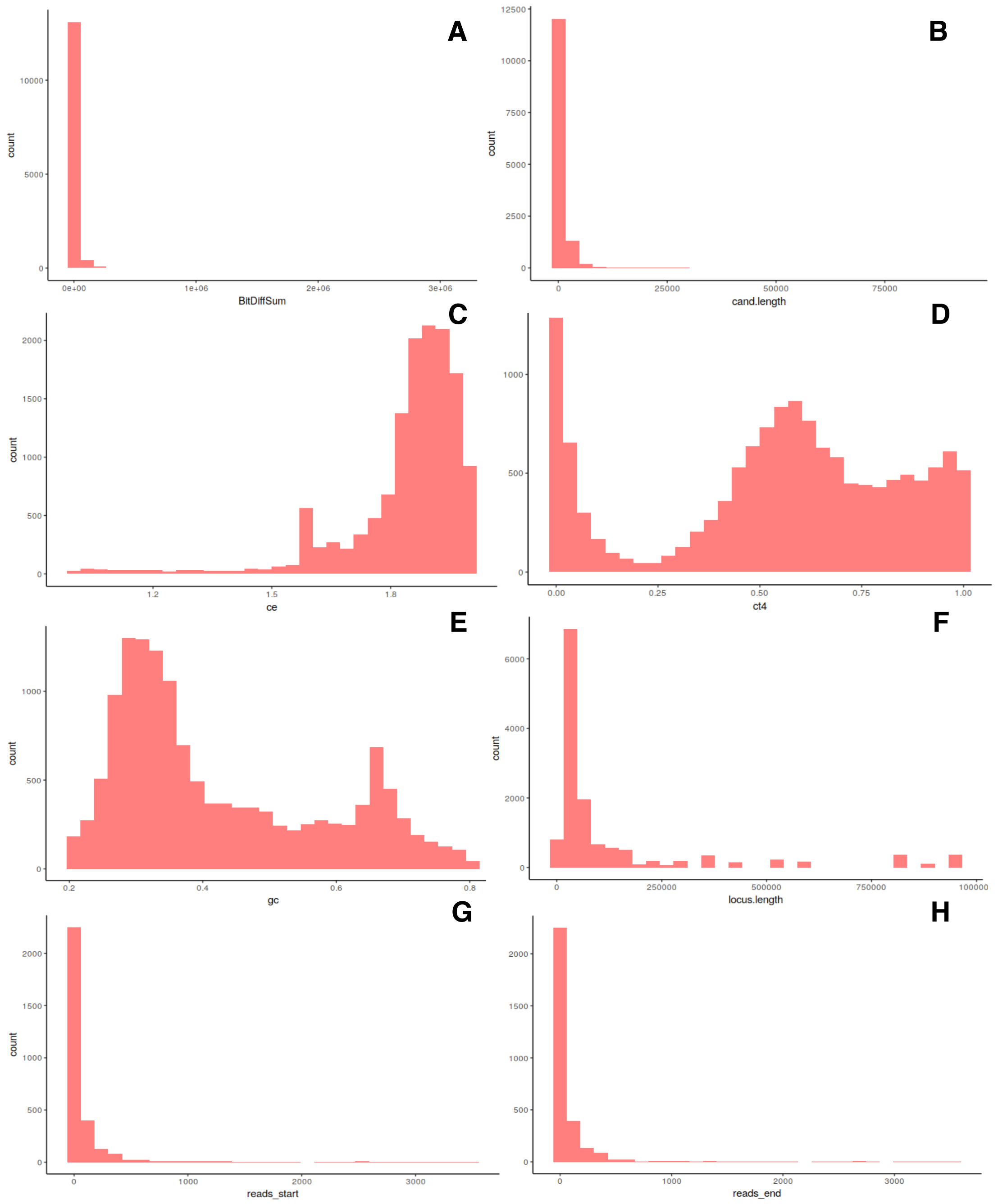

Figure S19. Parameters used for automated filtering of HGT candidates predicted by the HGT finder pipeline. Related to Material & Methods. Distributions of key filtering parameters used in the automated detection of HGT candidates, plotted as histograms with candidate counts on the y-axis. Each panel (A–H) represents one filtering criterion: (A) BitDiffSum (bitscore difference between prokaryotic and eukaryotic hits), (B) Candidate length (bp), (C) Sequence entropy (ce), (D) Trifnov's sequence complexity of order 4 (ct4), (E) GC content (%), (F) Genomic locus length (bp), (G+H) Number of reads overlapping the boundaries of the HGT. These distributions informed downstream filtering thresholds prior to manual curation.

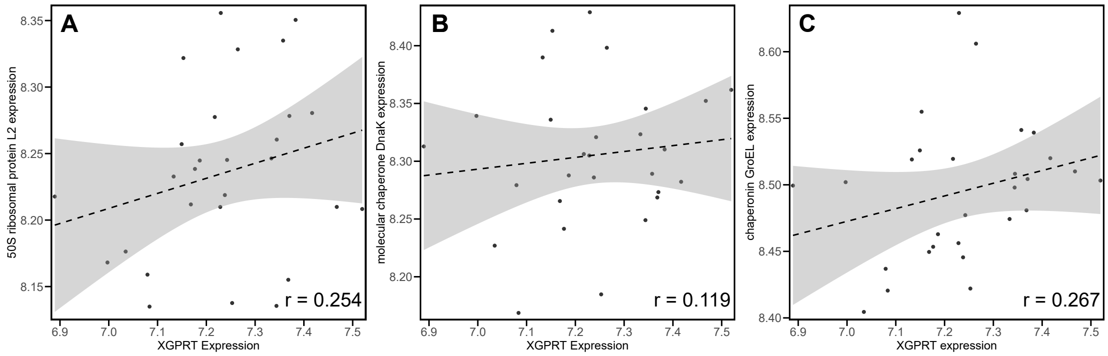

Figure S20. Testing for XGPRT correlation to *Westeberhardia* gene expression. Related to Material & Methods. To test whether the expression of the horizontally acquired XGPRT is associated with *Westeberhardia* titers in *C. obscurior*, we correlated XGPRT expression against expression of three *Westeberhardia* housekeeping genes. None of the genes correlated significantly with XGPRT (50S ribosomal protein L2, mean expression: 14.37 log2cpm, CV = 0.021, r = 0.254, adj. p-value = 1 (A); molecular chaperone DnaK; mean expression = 15.086 log2cpm, CV = 0.029, r = 0.119, adj. p-value = 1 (B); chaperonin GroEL; mean expression = 17.212 log2cpm, CV = 0.019, r = 0.267 (C)).

CobsLarvaeBActeriocytes_Green.pdf

512 x 512

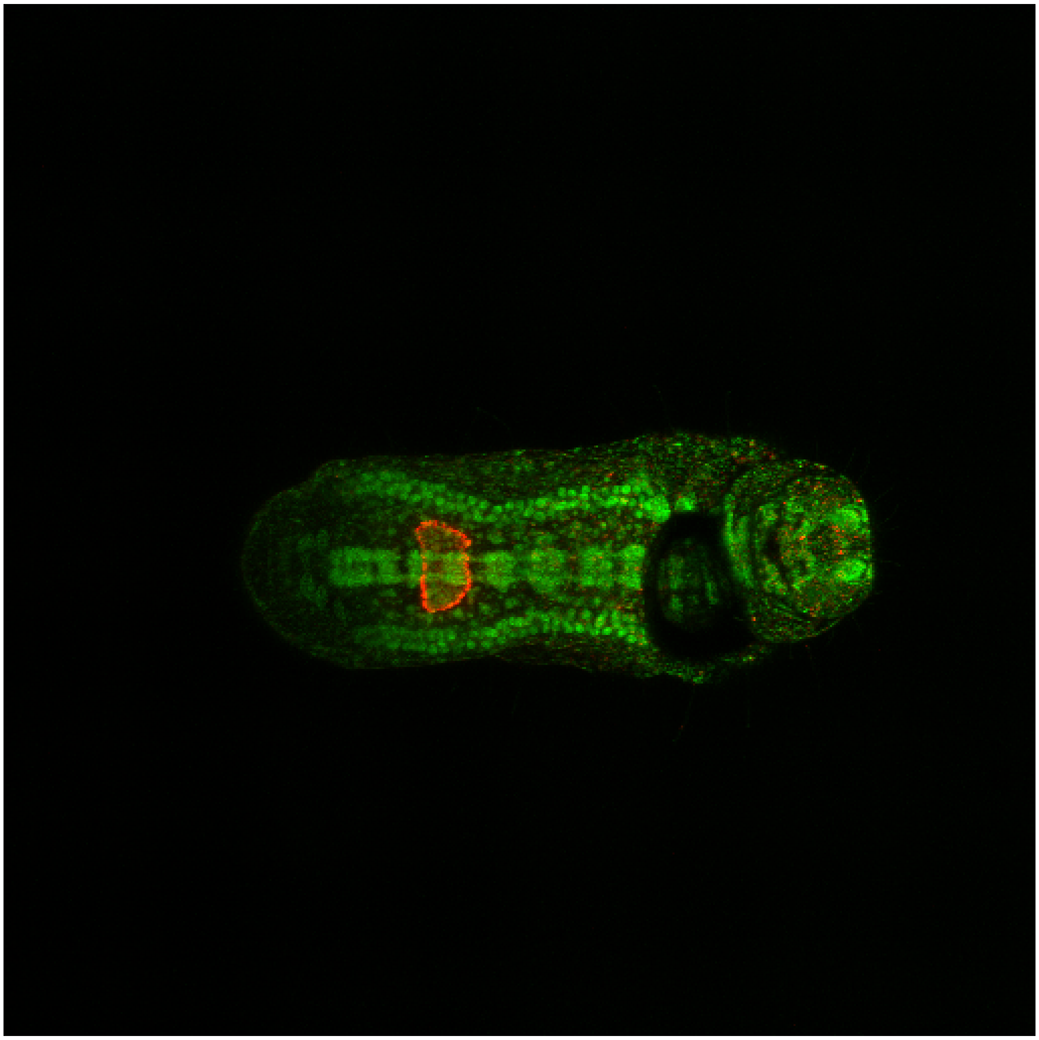

Figure S21. Representative HCR-FISH Image demonstrating low and diffuse background signal for the Alexa Fluor 647 in the absence of expression specificity. Related to Material & Methods.
