# Supplementary material for: Comparative analysis of 163 ant genomes reveals recurrent horizontal gene transfer from bacteria to ants
Source: Gigascience. 2026 Apr 6;15:giag043. doi: 10.1093/gigascience/giag043 (PMC13320244; doi:10.1093/gigascience/giag043)
Supplement: giag043_Authors_Response_To_Reviewer_Comments_Original_Submission [file giag043_authors_response_to_reviewer_comments_original_submission.pdf]

Author's Response To Reviewer Comments

Point-by-point response to Reviewer comments.

Reviewer: 1
Rinke and colleagues present a paper that looks at the integration of bacterial genetic material into ant genomes, using a previously published dataset. My expertise is in genomics, not (1) protein structure predictions and analysis or (2) function of the particular genes discussed in detail. That said, I think the writing and figures are overall excellent, and I have no major criticisms of this study. I enjoyed reading the paper, learned some new things, and think the genomic aspects for which I have expertise were quite well done.

Response:
We are very grateful to the reviewer for investing time and work to assess our manuscript. We have addressed all comments by Reviewer 1 in our revised manuscript, providing point-by-point responses below.

Reviewer: 1
I only have a few minor comments:

Is Blochmanniella the updated name for Blochmannia? (seen in recent papers about Blochmannia symbionts).

Response:
We thank the reviewer for pointing this out. The symbiont genus was originally described as "Candidatus Blochmannia"; but has indeed been recently updated to "Candidatus Blochmanniella" [1]. We have updated the terminology throughout the manuscript and clarified its synonymy at first mention.

1. Oren A. A plea for linguistic accuracy – also for Candidatus taxa. Int J Syst Evol Microbiol. 2017;67:1085–94. https://doi.org/10.1099/ijsem.0.001715

Reviewer: 1
Line 534: Figure S12 should be Figure S18.

Response:
We thank the reviewer for spotting this and have made the changes accordingly.

Reviewer: 1
In the left-hand side of Figure 2A, should K. nyos have a pink branch in the phylogeny as it is included in the alignment?

Response:
We agree with the reviewer that K. nyos should be highlighted in pink in the phylogeny on the left-hand side of Figure 2A, as it is included in the alignment, and we have updated the figure accordingly.

We note, however, that within this clade, P. punctatus does not possess the lysozyme HGT and is therefore not included in the alignment. This is now clarified in the revised figure legend, which states:

"The lysozyme HGT was not detected in the genome of P. punctatus, therefore this species is not included in the alignment of HGT sequences within this Crematogastrini clade."

Reviewer: 2
The study by Rinke et al re-analyzes an enormous number of ant genomes (related to Vizueta et al., 2025) with the goal of identifying horizontally transferred genes in a comprehensive manner across ants. The study is of interest and laudable in scope. Overall, the analyses were well-performed, the findings of

interest to the community, and the authors performed subsequent empirical testing of at least one candidate (XGPRT) which is often lacking from such work.

While this warrants publication I have several (relatively minor) comments that the authors should address before acceptance.

Response:
We would like to thank the reviewer for investing time and work to assess our manuscript. We have addressed all comments by Reviewer 2 in our revised manuscript, providing point-by-point responses below.

Reviewer: 2
What are the p-values given in figure 4G next to kegg terms? This does not appear to be clear from the text or legend.

Response:
We thank the reviewer for spotting this oversight. The provided values are FDR-corrected p-values from the hypergeometric tests performed for the KEGG enrichment analyses. We have clarified this in the figure legend, where we write:

“FDR values are Benjamini-Hochberg-adjusted p-values from KEGG pathway enrichment (hypergeometric test).”

Reviewer: 2
Why was read count > 100 used to delineate 'expressed' genes? This seems overly high if using normalized read counts (e.g. RPM) and library-specific in its utility if using raw read counts.

Response:
We acknowledge that our use of an absolute read count threshold requires clarification. To reduce computational time for the transcriptomic screens across all 130 species, we limited our analyses to the candidate HGT genes, mapping available RNAseq data against only the corresponding genomic regions, rather than to whole genomes or transcriptomes (with stringent alignment parameters, see Methods). Thus, normalization of read counts (e.g., RPM/TPM) could not be applied. Further, our primary goal was not to quantify relative expression levels, but rather to identify robust evidence of transcription of candidate HGT loci. We therefore applied a conservative threshold of >100 mapped reads to define expressed genes. This cutoff was chosen to minimize false positives arising from spurious or low-level background mapping and to ensure that only loci with clear transcriptional support were considered expressed.

We have added the following sentences to the Method section to make this explicit:

“As reads were mapped exclusively to candidate HGT regions rather than full genomes, we only report absolute read counts, using a threshold of >100 raw mapped reads as a conservative criterion to indicate robust transcriptional evidence of candidate HGT loci.”

Reviewer: 2
For figure 3, it would be good to show +/- 5kb from the shown genes as a supplemental panel/figure including any flanking genes on either strand. These genes show strange RNAseq coverage and in some cases substantial read pileup outside of the demarcated HGT locus (eg 3B, C and D), thus it would be good to show that the RNAseq data shows the expected low levels upstream of the putative HGT loci and also that these loci are not downstream of readthrough 3'UTRs of flanking genes. If these RNAseq data are stranded then including this information would further raise confidence given the strange very uneven coverage of RNAseq over these loci.

Response:
We agree with the Reviewer that examining the upstream and downstream regions regarding the RNAseq signal is a good addition. We have added Supplementary Figure S8 which contains IGV screenshots for

each of the six HGTs from Figure 3 showing the following tracks: RNAseq coverage data, general gene annotations from Vizueta et al. 2025, and the annotated HGT CDS loci. This analysis also revealed that for the murA HGT in Pheidole pallidula (Figure 3B), there is indeed overlap with unannotated exons on the reverse strand, that likely influence our estimate of the HGTs expression. We have therefore added the following sentence to the manuscript:

"..., and a […] (murA) in Pheidole pallidula (Fig. 3B). Note that RNAseq data indicate overlap of the murA CDS with an unannotated exon of gene Ppal_g15224 on the reverse strand (Supplementary Figure S21B), potentially causing an overestimate of the murA expression level."

We thank the Reviewer for the suggestion, allowing us to identify this issue.

Reviewer: 2
For Lasius flavus s1/2 are these distinct assemblies of the same species? Given the apparent dramatic difference in HGT genes between these two it would be good to speak to this, particularly given the differences in bacterial contamination reported between these samples in Vizueta et al., 2025.

Response:
Yes, these are two distinct assemblies of the same species, derived from independent samples from two different populations.

A display error in Figure 1 caused the species labels to be slightly misaligned, which may have given the impression that the two L. flavus variants differ substantially in their HGT gene content. We thank the Reviewer to make us aware of this mistake. We have fixed this issue in the revised Figure 1.

In Supplementary Table S1, we report 8 candidate HGT loci for L. flavus s1 and 11 for L. flavus s2. All 19 HGTs are putatively derived from Wolbachia in both assemblies and 17 of these 19 loci carry horizontally transferred Ankyrin repeat proteins. Our analyses demonstrated that these Ankyrin repeat proteins evolve particularly quickly, which likely explains the differences we observe for the two L. flavus variants.

Reviewer: 2
for HCR-FISH was a negative control probe used? This should be shown to validate signal specificity for positive results.

Response:
We thank the reviewer for this comment regarding signal specificity in the HCR-FISH experiments. Although a dedicated negative control probe was not included, several lines of evidence support the specificity of the observed signals.

First, the 16S rRNA probe designed to specifically target Westeberhardia endosymbionts effectively serves as an internal negative control in all tissues except the ovaries, as this endosymbiont is restricted to ovaries and bacteriomes in Cardiocondyla ants. Accordingly, no signal is detected in other tissues, indicating low background and high probe specificity. Second, the XGPRT mRNA signal is consistently localized within cells and not in luminal spaces, and no overlap between fluorescence channels was observed. This argues against nonspecific probe accumulation, bleed-through, or cross-reactivity.

In addition, we assessed nonspecific background signal in parallel HCR-FISH experiments using the same fluorophore (Alexa Fluor 647). These controls showed minimal background, which, when present, was diffuse and not spatially structured. In contrast, the signal observed in queen ovary samples is highly localized and spatially consistent with bacteriocyte-associated regions.

We have now included a representative image demonstrating the low and diffuse background signal in this channel (Supplementary Fig. 21). Together, these observations support the specificity of the HCR-FISH signal.

Cookie Preference Center

We use cookies which are necessary to make our site work. We may also use additional cookies to analyze, improve and personalize our content and your digital experience. For more information, see our Cookie Policy.

You may choose not to allow some types of cookies. However, blocking some types may impact your experience of our site and the services we are able to offer. See the different category headings below to find out more or change your settings.

Allow all

Manage Consent Preferences

Strictly Necessary Cookies

Always active

These cookies are necessary for the website to function and cannot be switched off in our systems. They are usually only set in response to actions made by you which amount to a request for services, such as setting your privacy preferences, logging in or filling in forms. You can set your browser to block or alert you about these cookies, but some parts of the site will not then work.

Functional Cookies

These cookies enable the website to provide enhanced functionality and personalisation. They may be set by us or by third party providers whose services we have added to our pages. If you do not allow these cookies then some or all of these services may not function properly.

Performance Cookies

These cookies allow us to count visits and traffic sources so we can measure and improve the performance of our site. They help us to know which pages are the most and least popular and see how visitors move around the site.

Targeting Cookies

These cookies may be set through our site by our advertising partners. They may be used by those companies to build a profile of your interests and show you relevant adverts on other sites. If you do not allow these cookies, you will experience less targeted advertising.

Cookie List

Clear

Apply Cancel
Consent Leg.Interest

Confirm my choices

Powered by onetrust
