# Supplementary material for: Comparative analysis of 163 ant genomes reveals recurrent horizontal gene transfer from bacteria to ants
Source: Gigascience. 2026 Apr 6;15:giag043. doi: 10.1093/gigascience/giag043 (PMC13320244; doi:10.1093/gigascience/giag043)
Supplement: giag043_Reviewer_2_Report_Original_Submission — Reviewer 2 -- 3/3/2026 [file giag043_reviewer_2_report_original_submission.pdf]

Reviewer Report

Title: Comparative analysis of 163 ant genomes reveals recurrent horizontal gene transfer from bacteria to ants

Version: Original Submission **Date:** 3/3/2026

Reviewer name: Reviewer 2

Reviewer Comments to Author:

The study by Rinke et al re-analyzes an enormous number of ant genomes (related to Vizueta et al., 2025) with the goal of identifying horizontally transferred genes in a comprehensive manner across ants. The study is of interest and laudable in scope. Overall the analyses were well-performed, the findings of interest to the community, and the authors performed subsequent empirical testing of at least one candidate (XGPRT) which is often lacking from such work.

While this warrants publication I have several (relatively minor) comments that the authors should address before acceptance:

-What are the p-values given in figure 4G next to kegg terms? This does not appear to be clear from the text or legend.

-Why was read count > 100 used to delineate 'expressed' genes? This seems overly high if using normalized read counts (eg RPM) and library-specific in its utility if using raw read counts.

-For figure 3, it would be good to show +/- 5kb from the shown genes as a supplemental panel/figure including any flanking genes on either strand. These genes show strange RNAseq coverage and in some cases substantial read pileup outside of the demarcated HGT locus (eg 3B, C and D), thus it would be good to show that the RNAseq data shows the expected low levels upstream of the putative HGT loci and also that these loci are not downstream of readthrough 3'UTRs of flanking genes. If these RNAseq data are stranded then including this information would further raise confidence given the strange very uneven coverage of RNAseq over these loci.

-For Lasius flavus s1/2 are these distinct assemblies of the same species? Given the apparent dramatic difference in HGT genes between these two it would be good to speak to this, particularly given the differences in bacterial contamination reported between these samples in Vizueta et al., 2025.

-for HCR-FISH was a negative control probe used? This should be shown to validate signal specificity for positive results.

Methods

Are the methods appropriate to the aims of the study, are they well described, and are necessary controls included? Choose an item.

Conclusions

Are the conclusions adequately supported by the data shown? Choose an item.

Reporting Standards

Does the manuscript adhere to the journal's guidelines on minimum standards of reporting? Choose an item.

Choose an item.

Statistics

Are you able to assess all statistics in the manuscript, including the appropriateness of statistical tests used? Choose an item.

Quality of Written English

Please indicate the quality of language in the manuscript: Choose an item.

Declaration of Competing Interests

Please complete a declaration of competing interests, considering the following questions:

- Have you in the past five years received reimbursements, fees, funding, or salary from an organisation that may in any way gain or lose financially from the publication of this manuscript, either now or in the future?
- Do you hold any stocks or shares in an organisation that may in any way gain or lose financially from the publication of this manuscript, either now or in the future?
- Do you hold or are you currently applying for any patents relating to the content of the manuscript?
- Have you received reimbursements, fees, funding, or salary from an organization that holds or has applied for patents relating to the content of the manuscript?
- Do you have any other financial competing interests?
- Do you have any non-financial competing interests in relation to this paper?

If you can answer no to all of the above, write 'I declare that I have no competing interests' below. If your reply is yes to any, please give details below.

I declare that I have no competing interests.

I agree to the open peer review policy of the journal. I understand that my name will be included on my report to the authors and, if the manuscript is accepted for publication, my named report including any attachments I upload will be posted on the website along with the authors' responses. I agree for my report to be made available under an Open Access Creative Commons CC-BY license (http://creativecommons.org/licenses/by/4.0/). I understand that any comments which I do not wish to be included in my named report can be included as confidential comments to the editors, which will not be published.

I agree to the open peer review policy of the journal

To further support our reviewers, we have joined with Publons, where you can gain additional credit to further highlight your hard work (see: https://publons.com/journal/530/gigascience). On publication of this paper, your review will be automatically added to Publons, you can then choose whether or not to claim your Publons credit. I understand this statement.

Yes Choose an item.
